# Supplementary material for: An optical neural network using less than 1 photon per multiplication
Source: Nat Commun. 2022 Jan 10;13:123. doi: 10.1038/s41467-021-27774-8 (PMC8748769; doi:10.1038/s41467-021-27774-8)
Supplement: Supplementary file 1 — Supplementary Information [file 41467_2021_27774_MOESM1_ESM.pdf]

Supplementary Materials for

An optical neural network using less than 1 photon per multiplication

Tianyu Wang,[1,*] Shi-Yuan Ma,[1] Logan G. Wright,[1,2]
Tatsuhiro Onodera,[1,2] Brian C. Richard,[3] and Peter L. McMahon[1,†]

[1] *School of Applied and Engineering Physics, Cornell University, Ithaca, NY 14853, USA*
[2] *NTT Physics and Informatics Laboratories, NTT Research, Inc., Sunnyvale, CA 94085, USA*
[3] *School of Electrical and Computer Engineering, Cornell University, Ithaca, NY 14853, USA*

CONTENTS

I Experimental Setup 3

Supplementary Note 1. Overview of the Experimental Setup and Components 3

Supplementary Note 2. Properties of the OLED Display 6

Supplementary Note 3. Intensity Modulation with a Phase-only SLM 6

Supplementary Note 4. Characterization of the Photodetector (MPPC) 7

Supplementary Note 5. Alignment of the Optical Imaging System 11

Supplementary Note 6. Correction of Optical Vignette 13

Supplementary Note 7. Pixel Walk-off and Crosstalk due to Imaging Imperfections 13

Supplementary Note 8. System Noise Characteristics 14

Supplementary Note 9. Optical Fan-in and Detection Energy Consumption 16

Supplementary Note 10. Relation to Stanford Matrix-vector Multiplier 19

II Vector-Vector Dot Product Accuracy 21

Supplementary Note 11. Computing Dot Products with Signed Elements using Incoherent Light 21

Supplementary Note 12. Characterization of Dot Product Accuracy with Varying Photon Budget 22
A. Generation of Test Datasets 22
B. Data Collection Scheme and Photon Budget Control 22
C. Calibration of Detector Readouts 23
D. Quantification of Single-Shot Dot Product Computation Error 23

* tw329@cornell.edu
† pmcmahon@cornell.edu

III Optical Neural Network for Image Classification 26

Supplementary Note 13. Training Protocol of Noise Resilient Optical Neural Networks 26

Supplementary Note 14. Workflow for Running Optical Neural Networks for Inference 27

Supplementary Note 15. Energy Efficiency of the Optical Neural Network 29

Supplementary References 33

Part I

Experimental Setup

Supplementary Note 1. OVERVIEW OF THE EXPERIMENTAL SETUP AND COMPONENTS

The optical vector-vector dot product multiplier setup consists of an array of light sources, a zoom lens imaging system, an intensity modulator, and a photodetector (Supplementary Figure 1). We used an organic light-emitting diode (OLED) display of a commercial smartphone (Google Pixel 2016 version) as the light source for encoding input vectors. The OLED display consist of a 1920×1080 pixel array, with individually controllable intensity for each pixel (for details, see Supplementary Note 2). A reflective liquid-crystal spatial light modulator (SLM, P1920-500-1100-HDMI, Meadowlark Optics) was combined with a half-wave plate (HWP, WPH10ME-532, Thorlabs) and a polarizing beamsplitter (PBS, CCM1-PBS251, Thorlabs) to perform intensity modulation as weight multiplication (for details, see Supplementary Note 3). The SLM has a pixel array of dimensions 1920×1152, with individually controllable transmission for each pixel. A zoom lens system (Resolv4K, Navitar) was used to image the OLED display onto the SLM panel (for details, see Supplementary Note 5). The intensity-modulated light field reflected from the SLM was further de-magnified and imaged onto the detector, by a telescope formed by the rear adapter of the zoom lens (1-81102, Navitar) and an objective lens (XLFLUOR4x/340, Olympus). An additional band-pass filter (BPF, FF01-525/15-25, Semrock) and polarizer (LPVISE100-A, Thorlabs) were inserted into the telescope (Supplementary Figure 1) in order to reduce the bandwidth and purify the polarization of the light reflected by the PBS, resulting in more precise results. During alignment and troubleshooting, we used a camera (Prime 95B Scientific CMOS Camera, Teledyne Photometrics) as a multi-pixel detector (Supplementary Figure 2a). For sensitive measurements under extremely low photon fluxes, we used a multi-pixel photon counter (MPPC, C13366 series GA type, Hamamatsu Photonics) as a bucket detector (Supplementary Figure 2b) (for details, see Supplementary Note 9). When it was necessary to further reduce the optical power, an additional neutral density filter (ND=0.4, NE2R04B, Thorlabs) was placed in front of the zoom lens to attenuate light.

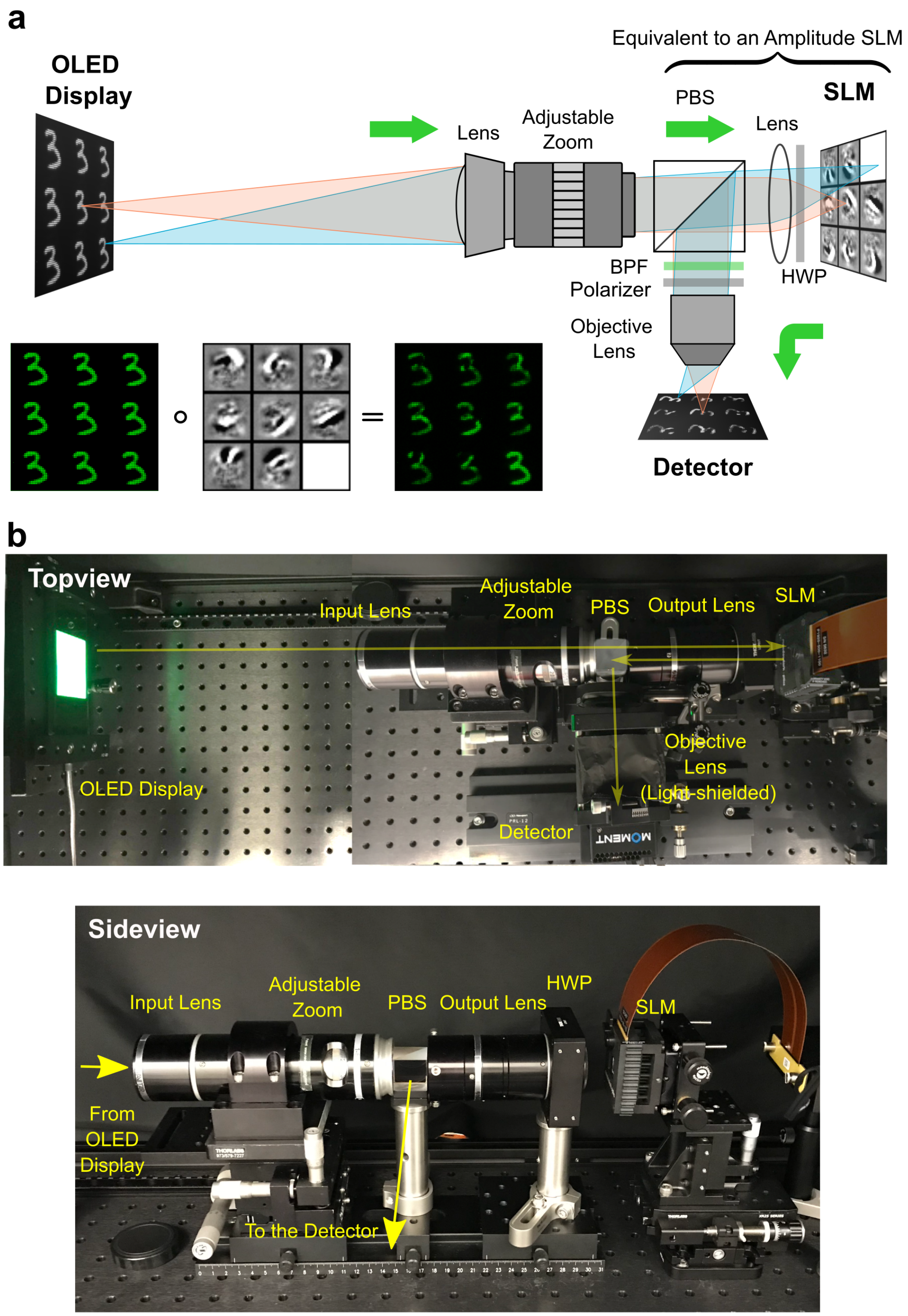

Supplementary Figure 1. Experimental Layout for the Optical Vector-vector Dot Product Multiplier Setup. a, The schematic of the optical setup. An illustration of the element-wise multiplication is also shown. In this example, nine copies of an input vector of handwritten digits, all '3'—which our setup accepts as 2D images—are intensity modulated by different weight vectors, which are each encoded as a 2D block on the spatial light modulator. Images of the vectors before and after the intensity modulation were taken by a camera placed at the detector location. (PBS: polarizing beam splitter; HWP: half-wave plate; BPF: band-pass filter; SLM: spatial light modulator) **b,** Photos of the core setup corresponding to the schematic are shown in panel (a).

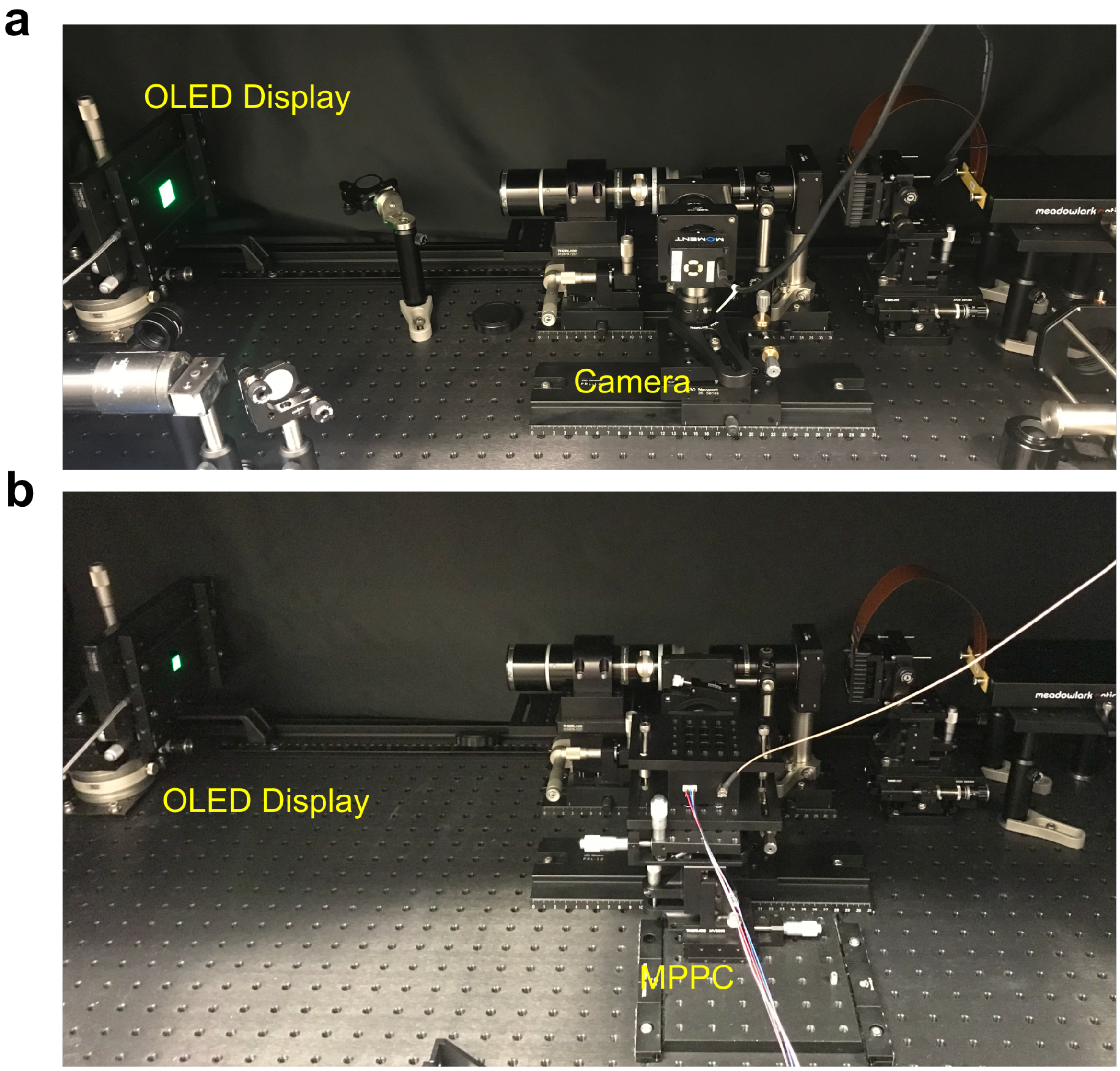

Supplementary Figure 2. Photos of the Entire Setup. a, The setup configured with a CMOS camera as a multi-pixel detector. **b,** The setup configured with an MPPC as a sensitive bucket detector.

Supplementary Note 2. PROPERTIES OF THE OLED DISPLAY

We chose an OLED display, which is made up of spatially and temporally incoherent light sources, to encode input vector $\vec{x}$ for several reasons. OLED pixels feature a high extinction ratio and high dynamic range in intensity, which are ideal for characterizing the accuracy of vector-vector dot products. A commercial-grade integrated OLED panel with a high pixel count is readily available at a low cost, which made it possible to encode very large vectors that were essential for demonstrating vector-vector dot products on our setup. Even though OLEDs are unable to encode phase information like coherent sources (e.g., lasers), our setup design based on optical imaging is compatible with both coherent and incoherent light.

Compared to other options of integrated incoherent light sources (e.g., liquid-crystal display, LCD), OLED pixels can be turned off completely, while LCD screens are always backlit with LED panels and thus always transmit some residual light. The true darkness of OLED pixels allowed us to achieve high dynamic range in intensity modulation and to reduce noise caused by background light pollution. Finally, the OLED display pixels used in this experiment were conveniently point-shaped and arranged in the same square lattice array as the SLM pixels (Supplementary Figure 3a), which facilitated pixel-to-pixel alignment (see Supplementary Note 5), and enabled us to unambiguously quantify the number of spatial modes, each performing one analog scalar multiplication. In contrast, commercial LCD pixels are typically arranged in bars, which require optical image transformation before they can be aligned to SLM pixels.

The OLED display used in our study had three different colors of pixels: red, blue, and green. We used only the green pixels, which form a 1080×1920 square lattice array ($\sim 2 \times 10^6$ total pixels) as shown in Supplementary Figure 3a. The pixel pitch was measured to be 57.5 µm. The maximum power of each pixel was measured to be $\sim$1 nW, emitted in a very wide angle ($>$ 60 degrees). Since the light emitted from the OLED screen had a rather broad spectrum, we used a band-pass filter (FF01-525/15-25, Semrock) to reduce the bandwidth in order to improve coherence for more precise and stable phase modulation by the SLM (Supplementary Figure 3b). The intensity of each individual pixel can be controlled independently with 256 (8-bit) control levels. However, since the actual output intensity was not linear with the pixel control level, we calibrated a linear look-up table (LUT) that contains 124 distinct intensity levels ($\sim$7 bits, Supplementary Figure 3c).

Supplementary Note 3. INTENSITY MODULATION WITH A PHASE-ONLY SLM

We converted a phase-only SLM into an intensity modulator with a half-wave plate (HWP) and a polarizing beam splitter (PBS). The SLM pixels are made of birefringent liquid crystal layers, whose refractive index can be tuned by applying voltage across them. By controlling the refractive index of extraordinary light, the SLM pixels introduce a phase difference $\phi_e - \phi_o$ between the extraordinary and ordinary light, whose polarizations are perpendicular to each other. When a PBS and HWP were placed in front of a reflective SLM, the light field passed the components twice, once during the trip towards the SLM and once after being reflected by the SLM (Supplementary Figure 1a). One of the functions of PBS was to separate the output from the input light: the input light (incident to the SLM) was horizontally polarized and transmitted by the PBS, while the output light (reflected from the SLM) was vertically polarized, and therefore reflected by the PBS. The other function of the PBS is to convert the polarization state of the output light to its amplitude: the light modulated by the SLM was in general elliptically polarized, controlled by the phase difference $\phi_e - \phi_o$. The amplitude of the light field (and intensity in this case too) was modulated by selecting only the vertical component of the SLM-modulated light at the output port of the PBS. The HWP was placed with its fast axis rotated 22.5 degrees from the extraordinary axis of the SLM such that the intensity transmission could be tuned from 0 to 100%. Supplementary Figure 4a shows the calculated relationship between the intensity transmission

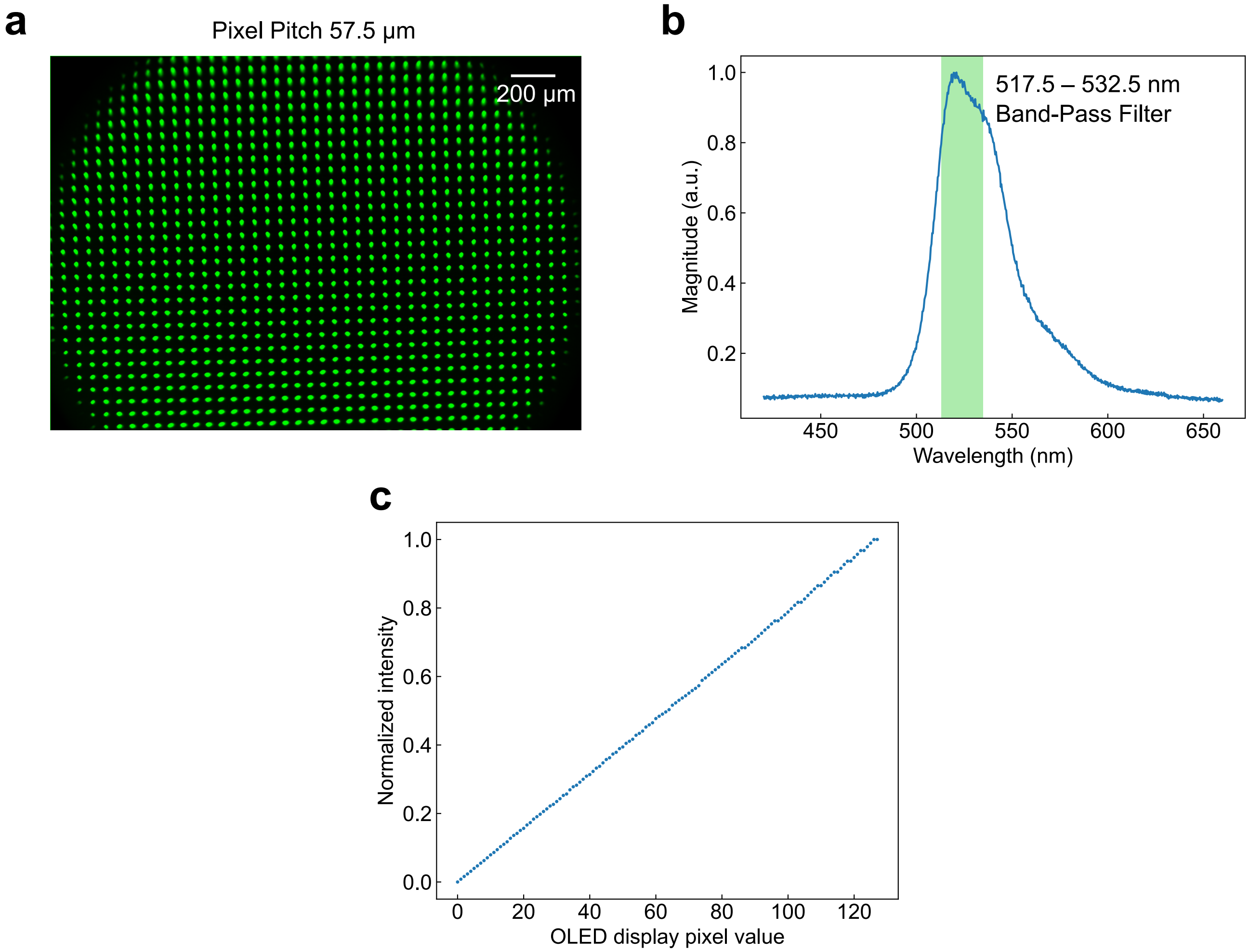

Supplementary Figure 3. Properties of the Organic Light-Emitting Diode (OLED) Display. a, An image of the green OLED pixels taken under an inspection microscope. The pixels form a square lattice with a pixel pitch of 57.5 μm. Scale bar, 200 μm. **b,** Emission spectrum of the green OLED pixels. The shaded area indicates the transmission band of the BPF ($> 90\%$ transmission). **c,** The 7-bit linear look-up table (LUT) calibrated to control the OLED display intensity.

and phase difference $\phi_e - \phi_o$. The maximum extinction ratio of the transmission intensity was measured to be $\sim$50 (Supplementary Figure 4b). The SLM consists of $1920 \times 1152 \sim 2.2 \times 10^6$ pixels, each of which can be independently controlled for intensity modulation with a 256 (8-bit) LUT (Supplementary Figure 4c). Alternatively, instead of using a phase-modulation SLM, the intensity modulator can be more compactly implemented with a monolithic LCD panel in a transmission geometry.

Supplementary Note 4. CHARACTERIZATION OF THE PHOTODETECTOR (MPPC)

For single-photon detection, we used a multi-pixel photon counter (MPPC) as a bucket detector. We chose an MPPC for its high signal-to-noise ratio (SNR), large measurement range, and moderately high bandwidth. The MPPC is composed of an array of Geiger-mode photodiodes with high intrinsic gain, which enables the photodiodes to detect single-photon events. The detection of each photon results in a spike-shaped impulse response in the output

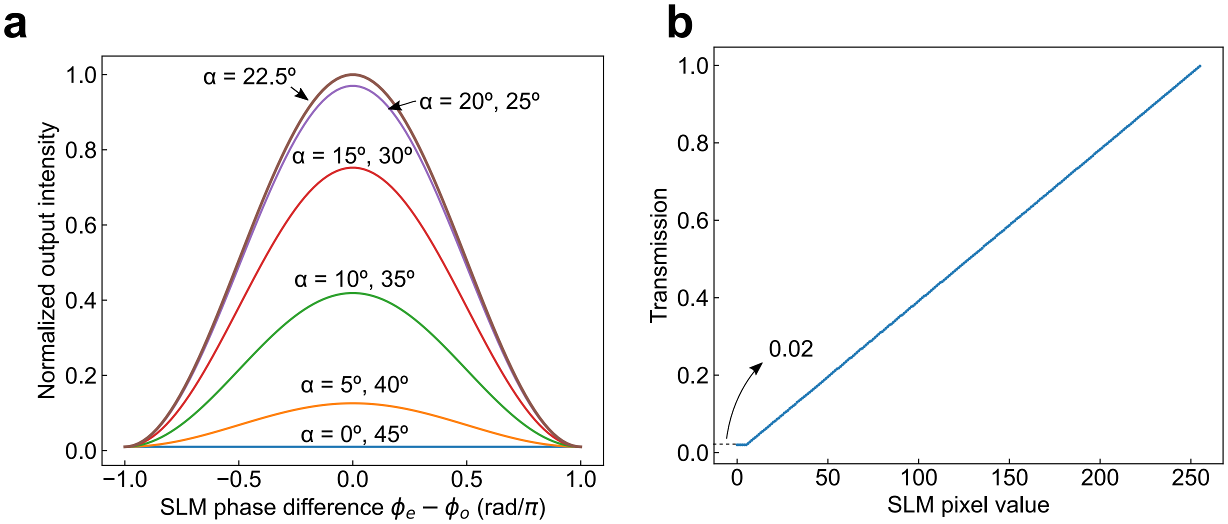

Supplementary Figure 4. Intensity Modulation with a Spatial Light Modulator (SLM). a, Simulation results of output intensity as a function of phase difference $\phi_e - \phi_o$. α is the angle between the half-wave plate (HWP) fast axis and the extraordinary axis of the SLM. **b,** The 8-bit LUT of the SLM for intensity modulation. The minimum transmission was measured to be ~0.02 times the maximum transmission, which is equivalent to an extinction ratio of ~50.

voltage of the detector, with a sharp rising edge and an approximately exponentially decaying tail (Supplementary Figure 5a).

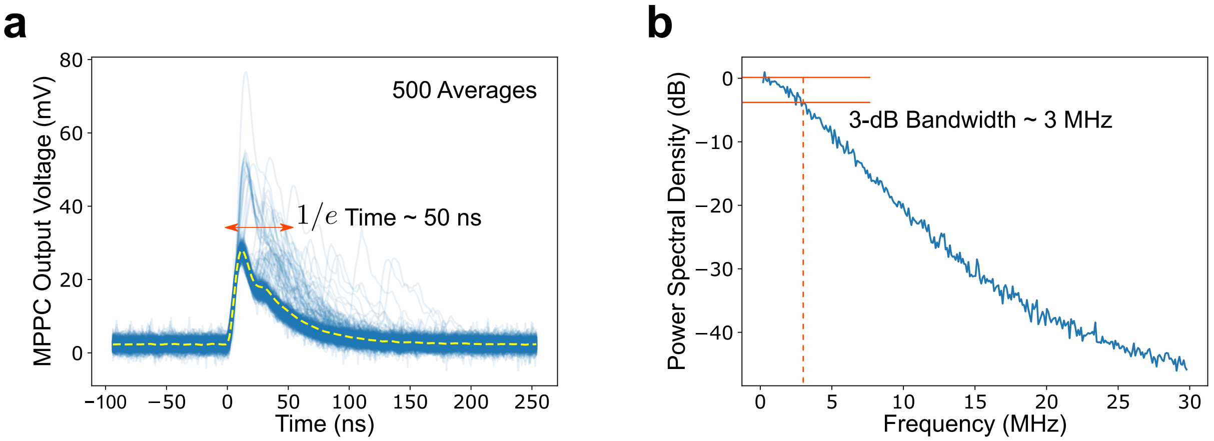

Supplementary Figure 5. Time and Frequency Characteristics of the Multi-Pixel Photon Counter (MPPC). a, The impulse response of single-photon detection averaged over 500 trials. **b,** The frequency response and Power Spectral Density of the MPPC.

When the photon flux rate is extremely low ($<10^7$ photons per second), the detected photons can be enumerated by counting the number of spikes (Supplementary Figure 6a). The maximum measurable photon flux rate is limited by the bandwidth of the detector (Supplementary Figure 5b) and potentially the dead time after each photon detection. To increase the maximum measurable photon flux (or optical power), the MPPC detector was spatially multiplexed with a 60×60 photodiode array (with $50\,\mu m \times 50\,\mu m$ pixel pitch), the outputs of which are then pooled into a single analog voltage trace. When the photon flux far exceeds the MPPC bandwidth ($\gg 10^7$ photons per second), the pulses

induced by individual photons overlap in time and can no longer be resolved (Supplementary Figure 6b). In this scenario, we measured the average output voltage, which maintains an excellent linear relationship with the average optical power impinging on the detector. The MPPC output voltage was calibrated against the power reading of a semiconductor power meter (818-UV-L-FC/DB, Newport), and the calibration result closely agreed with the manufacturer's specifications of the MPPC (Supplementary Figure 6d). Therefore, we were able to use the detector as a fast power meter to measure instantaneous optical power from pW up to several nW (Supplementary Figure 6d). In this experiment, optical power >6 pW was measured by converting the output voltage of the MPPC to the optical power impinging on the detector. Compared to regular semiconductor power meters without intrinsic gain—which can also measure ~pW levels of optical power—the MPPC can maintain a high SNR for a much higher bandwidth (~3 MHz, Supplementary Figure 5b), since its signal is amplified to overcome the noise integrated over the larger bandwidth.

When the MPPC is used as a power meter, the minimum power that can be measured is determined by the analog noise floor (including dark counts, thermal noise, and other electronic noises), which was measured to be equivalent to 1.25 pW optical power at the full bandwidth (Supplementary Figure 6c). The dark count of the MPPC was measured to be $\sim 10^4$ photons per second (<10 fW), which accounted for less than 1% of the total noise. Therefore, the detector can in principle measure optical power even below the analog noise equivalent power of 1.25 pW by means of photon counting. In our experiments, the photon counting measurement was conducted only to verify that the detector could indeed resolve single-photon events, and to determine the minimum valid optical power it could measure (~10 fW). Since the optical powers involved in our experiments were higher than the analog noise floor (~1.25 pW), they were all measured via the direct readout of detector's output voltage.

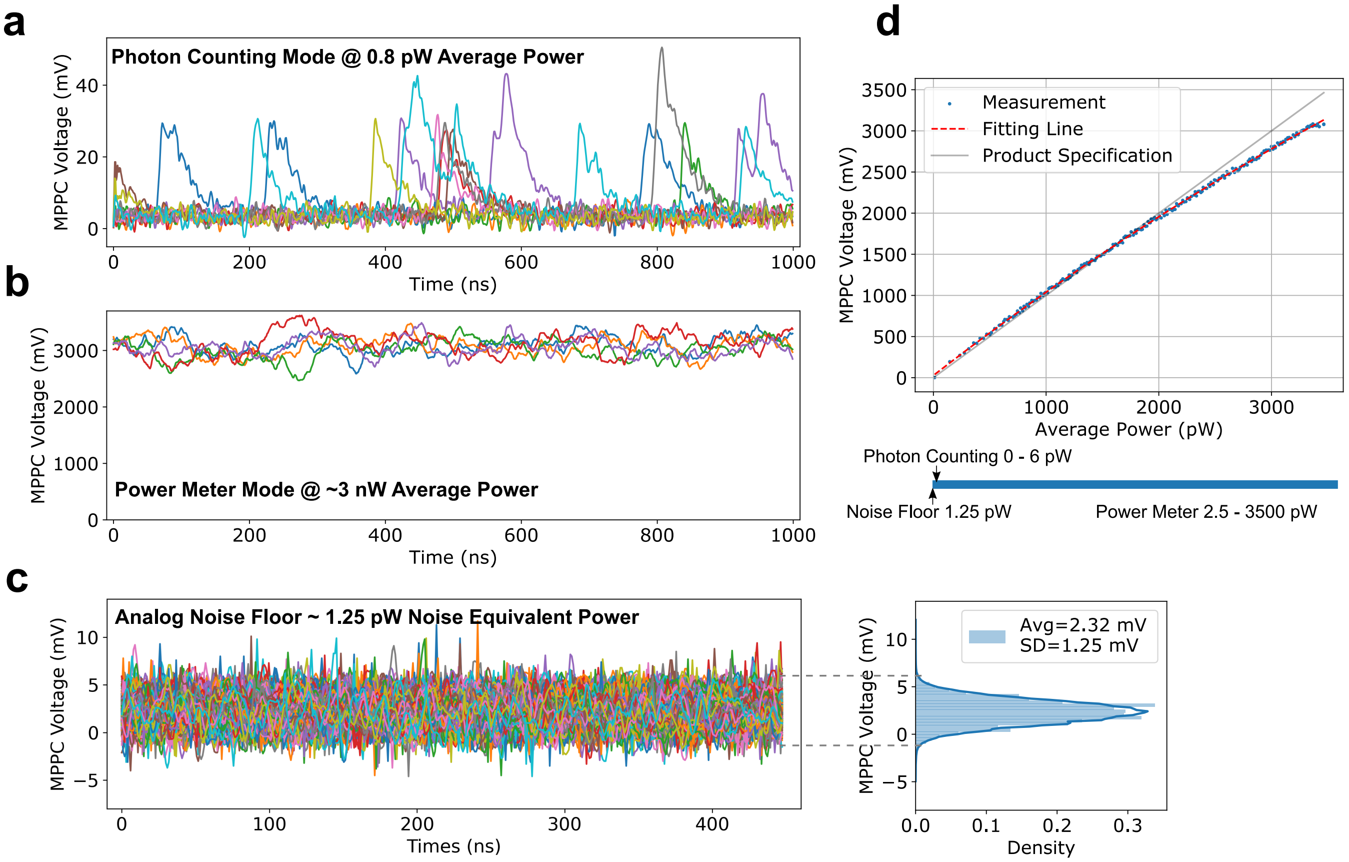

Supplementary Figure 6. Photon Detection and Optical Power Measurement with the MPPC. a, Single-photon detection under low photon flux. Different colors indicate instances of independent measurement trials. **b,** Instantaneous optical power measurement under high photon flux. **c,** The detector noise floor, which determines the lowest measurable optical power when the MPPC is used as a power meter. The left panel shows examples of independent measurements of the baseline analog noise; the right panel shows the noise distribution and statistics. **d,** The linear relationship between the average MPPC output voltage and the average optical power impinging on the detector. Calibration of the optical power (plot group "Measurement") was performed independently with a semiconductor power meter.

Supplementary Note 5. ALIGNMENT OF THE OPTICAL IMAGING SYSTEM

In order to maximize the vector-vector dot product multiplication size—and thus maximize the energy benefits of optical processing—we aligned as many pixels as possible from the OLED display to the SLM. Three conditions must be satisfied for this pixel-to-pixel alignment:

1. The OLED display must be imaged onto the SLM with a precise de-magnification factor to match the pitch of OLED pixels to that of the SLM pixels.

2. The imaging resolution needs to be high enough such that the image of each OLED pixel on the SLM must be no larger than the size of an SLM pixel. This is to prevent crosstalk.

3. The image of each OLED pixel must be aligned to the corresponding pixel on the SLM, which requires fine adjustment of the translation, rotation, pitch, and yaw of each device involved.

To match the pixel size of the OLED pixel image to the SLM pixel size, the zoom lens was set at a de-magnification of $9.2\,\mu m/57.5\,\mu m = 0.16$. This zoom factor was achieved when the zoom lens (Resolv4K 1-80100, Navitar) was configured with a $0.25\times$ lens attachment (1-81201, Navitar) and $1\times$ rear adapter (1-81102, Navitar). The zoom factor of the zoom lens could be mechanically tuned continuously to precisely match the OLED and SLM pixel pitch. Under this configuration, the spot size in the object plane of the zoom lens (the OLED side) is $40.85\,\mu m$ in diameter (Rayleigh criterion) according to the manufacturer's specifications. This spot size is smaller than the OLED pixel pitch size of $57.5\,\mu m$. Meanwhile, the spot size in the imaging plane (the SLM side) is specified to be $6.52\,\mu m$ in diameter (Rayleigh criterion), which is also smaller than the SLM pixel pitch size of $9.2\,\mu m$. In fact, the performance of the zoom lens system was close to the diffraction limit, and the images of OLED pixels on the SLM plane were well separated (Supplementary Figure 7). Therefore, the setup achieved the correct de-magnification factor and possessed adequate resolution, and both conditions (1) and (2) were satisfied.

To align each OLED pixel to the corresponding SLM pixel, mechanical alignment was performed using the following method (Supplementary Figure 7). First, identical images of the same size were displayed on both the OLED display and SLM. The bright pixels on the OLED display corresponded to pixels of full transmission on the SLM. The dark pixels on the OLED display corresponded to the pixels of zero transmission on the SLM. Therefore, the SLM functioned like a mask, with its light-transmitting parts identical in shape and size to the bright image on the OLED display. After intensity modulation by the SLM, the image on the OLED can only be preserved without any clipping if and only if the OLED and SLM pixels are exactly aligned to each other, and with the correct orientation (Supplementary Figure 7).

The maximum number of pixels that could be aligned was determined by the imaging error on the side of the field-of-view (FOV) of the zoom lens. According to the specifications of the zoom lens, at most 3.6 million OLED pixels in a square array can be aligned to the SLM. However, this estimation assumes diffraction-limited performance across the entire FOV. In practice, we managed to align $711 \times 711 \sim 0.5$ million pixels. There were three reasons for the deterioration of pixel-to-pixel alignment towards the side of the FOV:

1. Vignette: The optical transmission drops off towards the edge of the FOV, which causes up to a $\sim$90 % decrease in intensity for the 711×711 pixel array. As a result, the pixels around the center of the FOV must be dimmed in order to keep all pixels at the same brightness, as required for the computation of large dot products.

2. Image Distortion: nonlinear image distortion (such as barrel, or pin cushion, or other higher-order distortions) lead to a non-uniform local zoom factor of the OLED pixel image. Although linear image distortions can be corrected by mechanical alignment, nonlinear distortions cannot be completely fixed by alignment. Even slight distortions can cause pixels to slowly drift away from each other until eventually there is misalignment towards the side of the FOV.

3. Aberration: The aberration of the zoom lens increases towards the edge of its FOV and causes the focal spots to deviate from the diffraction-limited spot size. This causes the expansion of the OLED pixel image on the SLM plane, which couples part of the optical energy emitted from each OLED pixel into the surrounding SLM pixels that have incorrect transmissions for weight encoding.

The non-uniform transmission caused by the vignette could be fixed by making a pixel-wise LUT of the OLED display, which is described in Supplementary Note 6. While there is no easy solution to nonlinear image distortion and aberration, we characterize them in Supplementary Note 7.

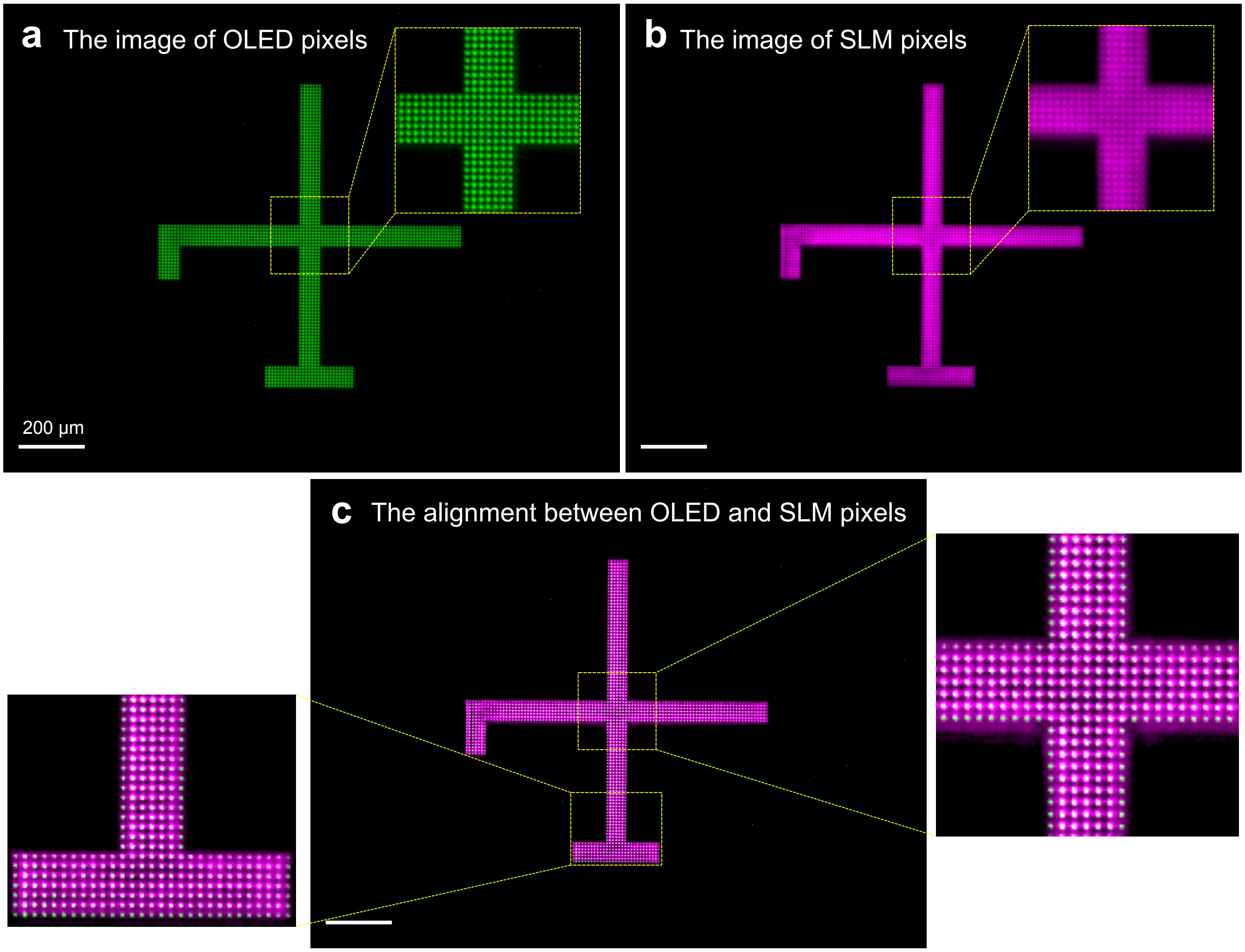

Supplementary Figure 7. Pixel-to-pixel Alignment Between the OLED and SLM Pixels. a, The image of a viewfinder pattern on the OLED display. The scale bar measures the distance on the SLM panel, 200 µm. **b,** The image of the identical viewfinder pattern modulated by the SLM. The entire SLM panel was uniformly illuminated by an ambient light source. The SLM performed intensity modulation which functioned as a mask with a hollow of the viewfinder shape. **c,** Visualization of the alignment between the OLED and SLM pixels.

Supplementary Note 6. CORRECTION OF OPTICAL VIGNETTE

To enable computation of large dot products, we corrected for intensity fall-off towards the edge of the FOV, caused by optical system vignettes as discussed in Supplementary Note 5. The correction was especially important for optical fan-in (discussed in Supplementary Note 9), since each pixel, regardless of its position in the FOV, should contribute the same amount of optical energy to the detector, if set at the same pixel value.

Correction was performed by making an attenuation map that compensates for different transmissions of pixels at different locations. We first configured the OLED to display at the maximum pixel brightness uniformly across the entire FOV, and then captured an image of the OLED display at the detector plane. Due to the vignette effect of the optical system, the intensity distribution was not uniform at the detector plane (Supplementary Figure 8, top left panel). A region of interest (ROI) was then chosen, in which we sought to achieve uniform intensity for all OLED pixels. The minimum intensity in the ROI was set as the target intensity value (circled areas in the top left panel of Supplementary Figure 8). The brightness of other OLED pixels was reduced iteratively until the intensity of their images matched that of the target value. The result of the correction is shown in Supplementary Figure 8, top right panel, where uniform intensity was achieved in an ROI of size 720×720. Meanwhile, an attenuation map was established to determine the percentage by which each OLED pixel should be attenuated in order to achieve a uniform output intensity.

Supplementary Note 7. PIXEL WALK-OFF AND CROSSTALK DUE TO IMAGING IMPERFECTIONS

We examined two aspects of the optical system's imaging quality: walk-off of pixel alignment due to image distortion, and degradation of focal spots due to aberration (both discussed in Supplementary Note 5). To visualize these effects, we captured an image of a sparse 2D grid of pixels displayed on the OLED screen. The grid was composed of blocks with an edge length of 80 pixels, with only the pixel at the center of each block was turned on. Supplementary Figure 9a shows how the imaging quality of single pixels changed across an ROI of 720×720 pixels. For example, images of a single pixel tended to be sharp and focused near the center of the FOV, while images of pixels towards the corner of the FOV spread out into a streak along the radial direction. This is probably due to coma aberration, which is common to most imaging systems (Supplementary Figure 9a). Meanwhile, the walk-off of pixel alignment could be observed by the deviation of the focus from the center of each block (Supplementary Figure 9a). As discussed in Supplementary Note 5, pixel walk-off due to linear image distortion can be corrected with careful mechanical alignment, while nonlinear distortion cannot be eliminated. Based on Supplementary Figure 9a, the pixel walk-off was insignificant for the ROI of 720×720. Incidentally, the largest possible ROI for optical matrix-vector multiplication was determined by the trade-off between optical power transmission and imaging quality distribution (Supplementary Figure 8 and Supplementary Figure 9a). Since the imaging quality was better on the right side, the ROI was shifted slightly to the right of the brightest part of the intensity distribution.

Pixel walk-off and crosstalk both resulted in errors in weight modulation, due to the coupling of optical energy into neighboring SLM pixels with incorrect modulation weights. These effects were quantified and modeled as so-called crosstalk kernels, which are similar to convolution kernels but vary gradually in space. Supplementary Figure 9b, c show the intensity distribution of a focal spot near the center (corner) of the FOV in a 7×7 block of SLM pixels, with the central pixel denoting the SLM pixel that the bright OLED pixel should align to. When both input vectors and weights were natural images (whose pixel value variation was smoother, and usually constitute the first layer of neural networks for image classification), the error caused by walk-off and crosstalk was less severe. For applications in optical neural networks (ONNs), such imaging errors were modeled during the training process by random affine transforms and 2D convolution to enhance the model's resilience to imaging errors (Supplementary Note 13).

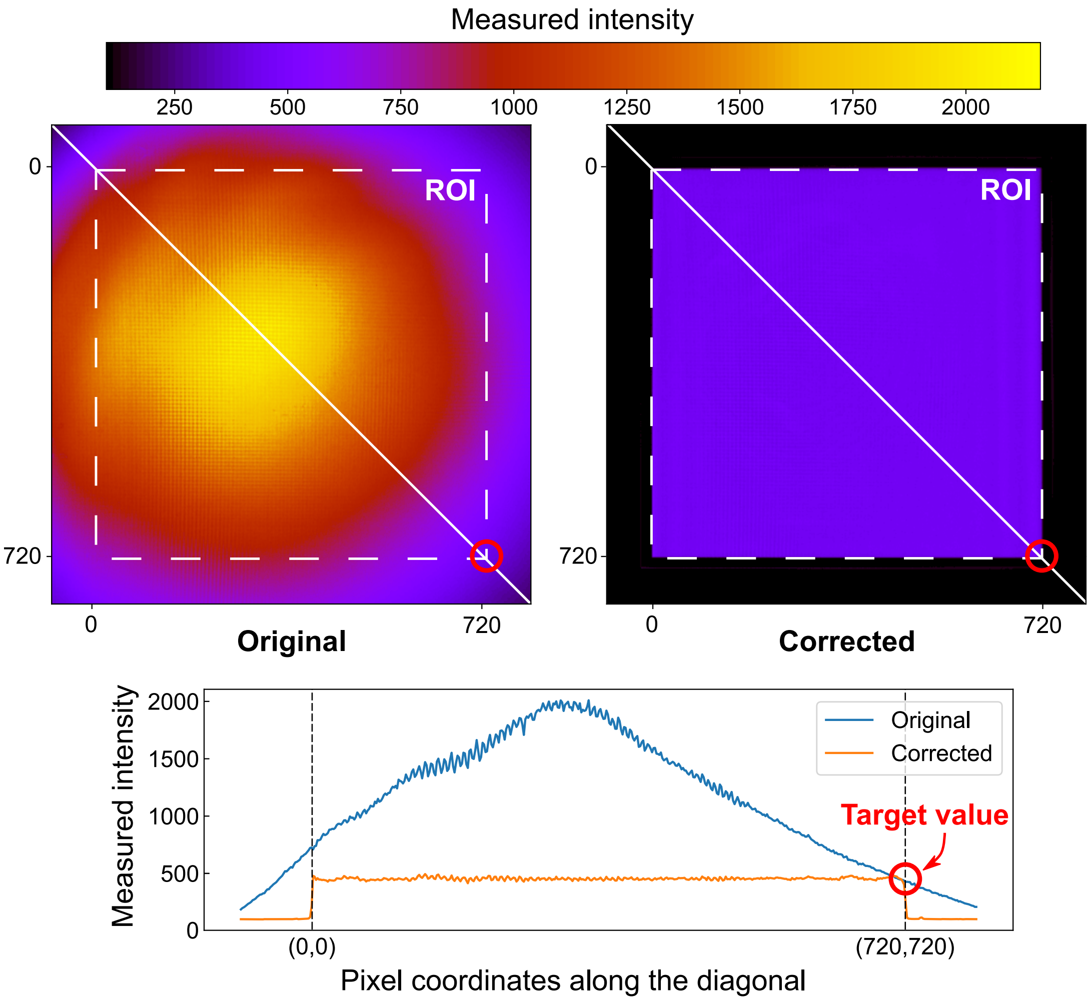

Supplementary Figure 8. Correction of Non-uniform Transmission of the Optical System. The original intensity distribution is shown in the top left panel ("Original"), with intensity falling off towards the edges of the region of interest (ROI). The correction procedure reduced the intensity of pixels near the center to match the target value near the darkest corner (720, 720) in the selected ROI. The image after correction is shown in the top right panel ("Corrected"). The bottom panel shows intensity along the diagonal pixels of the ROI (solid white lines) before and after correction.

Supplementary Note 8. SYSTEM NOISE CHARACTERISTICS

We examined temporal fluctuations of each part of the system and describe hindrances in approaching shot noise-limited performance. Overall, when the OLED was set at a constant brightness and the SLM at a constant transmission across all pixels, the SNR of optical power measurements were about half the shot noise-limited SNR (Supplementary Figure 10). Sources of excess noise, in addition to shot noise, include intensity fluctuation of the OLED display, phase instability of the SLM, and the intrinsic noise of the detector. At high optical power, noise from external sources dominates the SNR measurement; as the power decreases, shot noise becomes a dominant source of noise. At extremely low optical power, the intrinsic noise of the detector is mainly responsible for deviations from the shot noise-limited performance.

There are three main components causing intensity fluctuations in OLED displays: raster scanning during screen refreshing, pulse width modulation for brightness control, and the thermal noise of OLEDs. When a stationary image

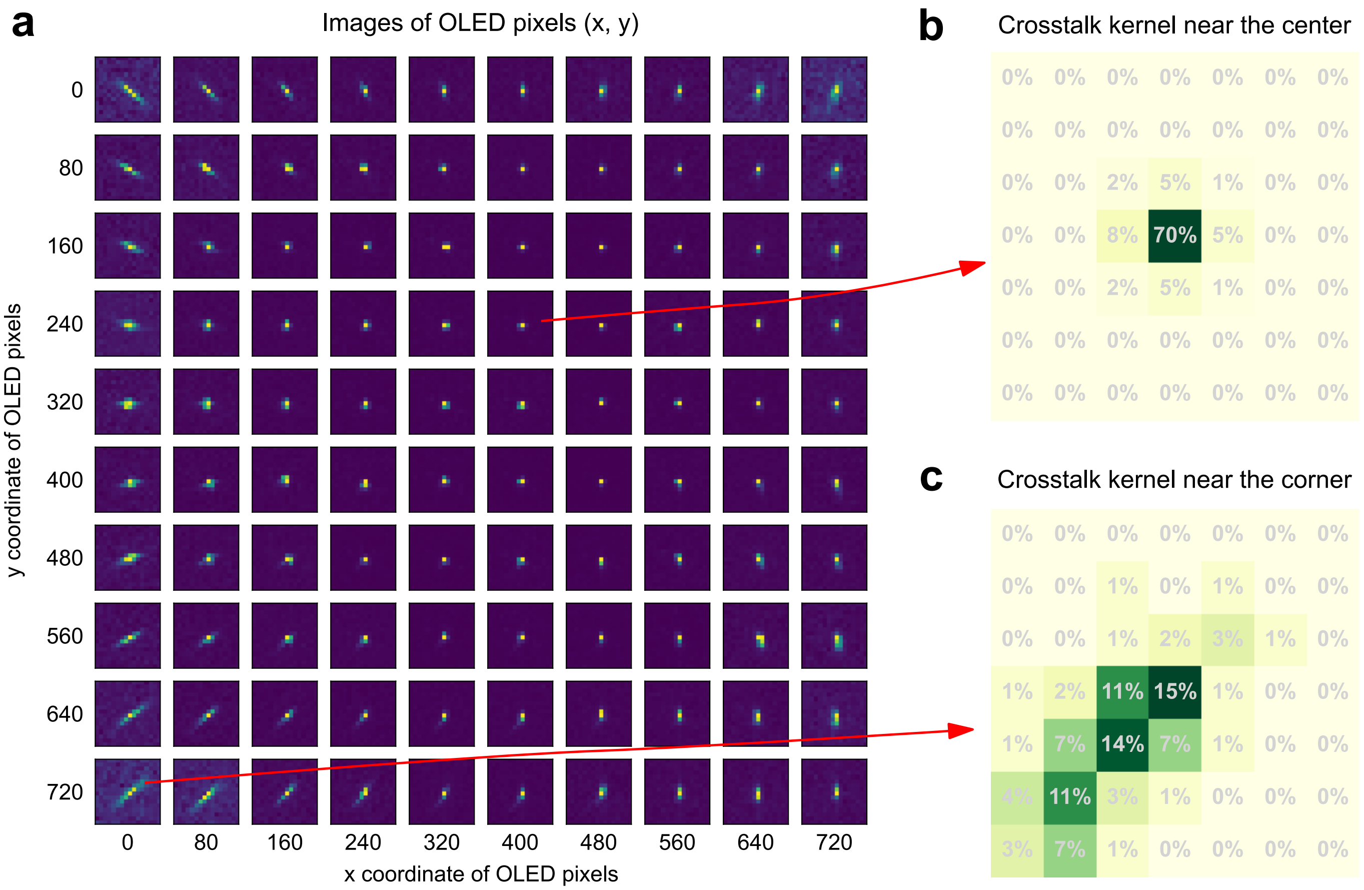

Supplementary Figure 9. Imaging Quality of the OLED Pixels. a, Images of the OLED pixels in a region of interest (ROI) of size 720×720. Only the pixels on the grid points of a 2D grid (with edge length of 80 pixels) were switched on. The OLED display was first imaged onto the SLM, and then relayed to the detector plane where the images were captured by a camera. The intensity inside each block was normalized to the maximum pixel value in the block. **b(c),** The 7×7 crosstalk kernels near the center (corner) of the FOV. Each entry denotes the percentage of optical power coupled into each individual SLM pixel, with all the power supposed to couple to the central pixel.

was shown on the OLED display, no perceivable raster scanning pattern was observed in high-speed videos of the display. There were occasional black scanning stripes and short bursts of flashing, which are likely to have been caused by some refreshing mechanism. The OLED display did not seem to use pulse width modulation to adjust brightness until very low brightness settings were reached (below $\sim$35 %). Therefore, we avoided setting pixels to low values, and instead used neutral density filters to attenuate light for extremely low-light measurements. The intensity fluctuation of the light source was mitigated by the high attenuation of the imaging system. Due to the large emission angle of OLED pixels ($>$ 60 degrees), most of the optical power was not collected by the zoom lens, which has a small collection angle. It was estimated that only $\sim$0.7 % of light was collected by the zoom lens (Supplementary Figure 11). The high loss converted the thermal state of the OLED light closer to a coherent state by coupling vacuum states to it [1], which improved the SNR and brought the ratio closer to the shot noise limit. It should be noted that even though the light collection efficiency was low for the zoom lens, the transmission from before the SLM to the detector (where the computation took place) was quite high at $\sim$22 % (Supplementary Figure 11). Thus, optical energy efficiency can be drastically improved if the OLED and zoom lens are replaced with a stable coherent source and an optical system with high transmission in a customized setup.

The phase fluctuation of the SLM stems from the constant switching of voltage across the liquid crystal layers. This

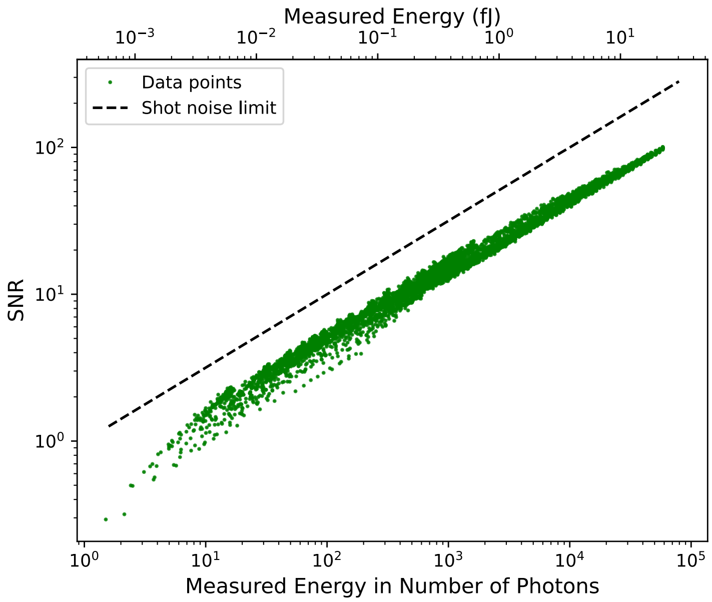

Supplementary Figure 10. The Signal-to-noise Ratio (SNR) as a Function of Photon Flux, as Measured by the MPPC. The integration window was 150 ns under analog mode.

fluctuation could be measured by monitoring the intensity fluctuation of the laser diffraction pattern generated by a phase grating on the SLM. According to the manufacturer, the SLM used in this experiment oscillated at 53 kHz, with a measured peak-to-peak power ripple of 0.24% for the first-order diffraction spot. Compared to OLED intensity fluctuations, instabilities caused by the SLM are relatively minor.

The intrinsic noise of the detector (e.g., thermal noise or dark counts) contributed to excess noise that was only apparent when the optical power to be measured was extremely weak. As discussed in Supplementary Note 4, the detector's intrinsic noise was negligible for high optical power ($\gg$1 pW). The analog noise floor becomes significant for low optical power at $\sim$1 pW, which necessitates photon counting for even lower photon flux.

Supplementary Note 9. OPTICAL FAN-IN AND DETECTION ENERGY CONSUMPTION

In the main text, we discussed how optical fan-in plays an important role in noise reduction during optical dot-product computation. Noise reduction is possible through the aggregation of a large number of terms in the element-wise vector-vector product. Since the signal-to-noise ratio is almost determined by the total photons required for a desired output, as vector size increases, the number of photons *per multiplication* decreases. Therefore, for extremely large vector sizes, it is possible to use even less than one photon per multiplication. In this section, we focus on other technical aspects of the optical fan-in operation, including its working principles, additional reasons why it has energy consumption benefits over digital accumulation operations, and generalization to other ONN platforms.

In this experiment, the optical fan-in operation carries out the summation in vector-vector dot product computation by focusing optical spatial modes onto the active area of a detector. More generally, equivalent operations (e.g., weighted optical fan-in, weighted sum of neural activation) exist universally in different spatial-domain optical processing schemes, including the Stanford matrix-vector multiplier [2], volume holography [3], $4f$ convolution [4, 5], and diffractive neural networks [6, 7]. In these schemes, each detector unit reads out the amount of optical energy contained in multiple superimposed spatial modes. The intensity or amplitude of each spatial mode can represent a scalar of some up-stream element-wise product between vectors. Therefore, the energy benefits achieved with optical

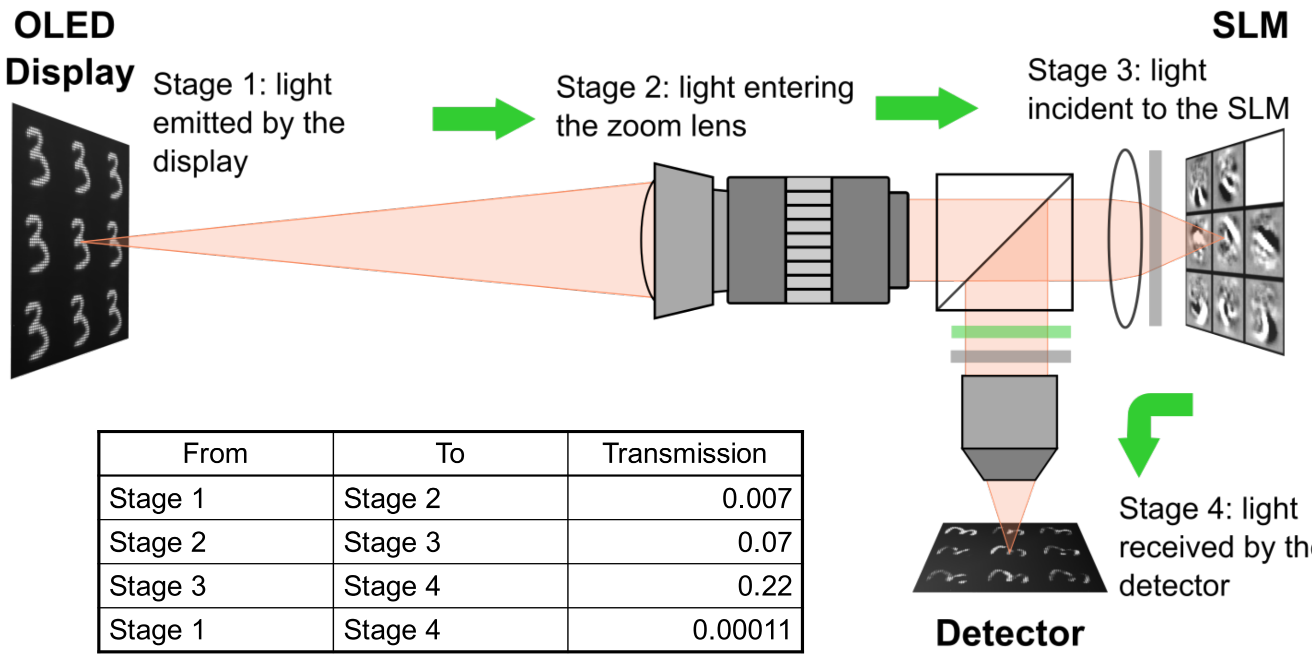

From	To	Transmission
Stage 1	Stage 2	0.007
Stage 2	Stage 3	0.07
Stage 3	Stage 4	0.22
Stage 1	Stage 4	0.00011

Supplementary Figure 11. The Optical Power Transmission at Each Stage of the Setup. A breakdown of the transmission of each stage of the experimental setup is listed in the inset table of the figure.

fan-in are potentially applicable to different spatial domain optical computing schemes, and the energy advantage would improve with the number of spatial modes summed on each single detector provided the noise of each spatial mode is statistically independent of each other.

Compared to digital summation, which sums the readouts from different detector pixels with digital electronic circuits, optical fan-in implements summation through the accumulation of photoelectrons generated in the same piece of detector material. Optical fan-in reduces energy consumption by skipping the digital summation circuits and reducing the number of pixels, along with their associated amplification circuits. Furthermore, compared to analog electronic summation via pooling of all the photoelectrons generated across a large-area detector, optical fan-in allows all the photoelectrons to be generated in the vicinity of a small-area detector simply by focusing light. In such a way, the energy associated with charging and discharging the detector can be further reduced by shrinking the size as well as the capacitance of the detector. Incidentally, to achieve this effect, the operation of optical fan-in does not require the combination of spatial optical modes.

Optical fan-in not only results in energy savings during the accumulation operation, but also reduces the energy costs of digital memory used for storing intermediate results. In the operation of typical neural networks, memory-associated energy costs often account for the majority of the total energy cost, exceeding even the energy spent on arithmetic operations [8]. Despite attempts to improve energy efficiency in digital electronic processors through the tailoring of data flow to machine-learning tasks [8] or incorporation of more on-chip memory [9], there is still a significant amount of memory that can theoretically be further reduced. For example, in systolic arrays, vector-vector dot products are computed by adding $w_i x_i$ ($i = 1...N$) term-by-term to a partial sum, with each term requiring N dedicated memory units in order to read and write a partial sum only once. Digital electronic adders usually have a small number of input operands, and cannot perform the summation of a large number of terms without saving the intermediate results. In comparison, optical fan-in can physically implement summation of a large number of terms (10^3-10^5) in a single step, which exempts the use of any intermediate memory units, potentially leading to substantial energy savings.

In this experiment, optical fan-in was performed by an objective lens, which projected a de-magnified image of the SLM onto the active area of the MPPC. The de-magnification factor from the SLM to the detector was 0.276×, and the total de-magnification factor from the OLED display to the detector was $\sim 0.0442\times$. In other words, each OLED pixel of original pitch 57.5 µm was imaged to a size of 2.54 µm on the detector. Therefore, the entire 711×711 pixel array could be projected onto the detector within a square of size $1.806\,\text{mm} \times 1.806\,\text{mm}$, which fits comfortably within the $3\,\text{mm} \times 3\,\text{mm}$ active area of the MPPC. During ideal optical fan-in, all spatial modes associated with a single dot product should be integrated by a single piece of detector material. In this experiment, since the MPPC consists of multiple photodiodes, the spatial modes were not all able to be focused onto a single photodiode (however, it should be noted that the photocurrents of these pixels were still summed as analog signals to form a single output, and no digital operations were involved). Each photodiode covered $(50\,\mu\text{m}/2.54\,\mu\text{m})^2 \sim 388$ spatial modes for in-pixel summation. Even with so many spatial modes, no saturation was observed. This is because the photon flux was extremely low for each spatial mode, and thus the simultaneous arrival of multiple photons was rare. The photoelectrons generated by different photodiodes were superimposed as a single analog voltage signal at the detector output.

The energy consumption of optical fan-in can be calculated based on the power consumption of the detector, and can be viewed as the energy cost of analog summation/accumulation. In this experimental setup, the MPPC works at a typical wall-plug power of 1.1 W. Each photodiode consumes, on average 0.3 mW, after dividing the wall-plug power by the total number of the photodiodes (3,600). During a short detector integration time of 100 ns, each photodiode consumes $0.3\,\text{mW} \times 100\,\text{ns} = 30\,\text{pJ}$ per readout cycle. Dividing this by the number of spatial modes captured by each photodiode yields the energy consumed for each addition. In this experiment, the minimum detector energy consumption of adding one spatial mode to the dot product sum was calculated to be $30\,\text{pJ}/388 =$ **77 fJ**.

In principle, the energy budget for each addition can be further reduced via a number of means: improving the optical fan-in implementation, using smaller focal spots or lower detector gain, and increasing the number of spatial modes for in-pixel summation. Here, we review in detail the effects of each strategy:

1. The focal spot can be reduced to $\lambda/2$ in air, based on the diffraction limit (Abbe's resolution limit with a numerical aperture of 1), which makes each spatial mode occupy $(\lambda/2)^2 = (532\,\text{nm}/2)^2 = 0.071\,\mu\text{m}^2$ area on the detector. The energy consumption for each addition scales with area, thus reducing the focal spot by half would require only $77\,\text{fJ} \times (0.532\,\mu\text{m}/2/2.54\,\mu\text{m})^2 = 0.84\,\text{fJ}$. In addition, the area of each spatial mode can be further reduced by focusing light in materials with refractive index larger than 1 (e.g., glass).

2. In this experiment, the photodiodes were operated with a high gain (Geiger mode) to provide extremely high SNR for single photon detection. In practice, a lower gain could provide two benefits. First, a lower gain can further reduce the energy cost per addition. A lower gain can also prevent saturation of the detector, and allows the accumulation of more terms in a dot product, which is essential for an optical energy advantage. Ideally, the detector volume should be minimized to reduce capacitance, which in turn reduces thermal noise and potentially allows for a high voltage across the detector, i.e. exempting the use of amplifiers [10, 11], Meanwhile, the number of carriers scales with the detector volume, which results in a limited full-well capacity for a small detector volume. A high gain results in each photoelectron being amplified into many electrons and quickly depletes the carriers in the detector volume. For this reason, the gain of the detector should be reduced until just enough to amplify the signal to overcome thermal noise. For a detector area of only one spatial mode (assuming $\lambda/2$ focal spot size of 525 nm), the RMS value of thermal noise electrons is calculated as $\sqrt{kTC} = \sqrt{4.14 \times 10^{-21}\,\text{J} \times 27.5\,\text{aF}} = 3.38 \times 10^{-19}\,\text{C} = 2.1e^-$ at room temperature [10, 11]. At a detection level of 1 photon per spatial mode, a moderately low gain can be applied such that the number of electrons generated by each photon is just large enough in comparison to the number of noise electrons. Such an optimization strategy has also been mentioned in Ref. [12].

3. The SNR of photon detection can also be improved by increasing the number of spatial modes for in-pixel

summation. Even though both detector area and capacitance scale with the number of spatial modes to be summed (N), the overall noise scales with $\sqrt{N}$ while the total signal photons scale with N. For example, with 0.5 signal photons (without any detector gain) and 2 noise electrons per spatial mode, the SNR = 0.25 for each spatial mode; with 10,000 spatial modes, the SNR can be enhanced to $0.25 \times \sqrt{10,000} = 25$. Therefore, even in the thermal noise-limited (as opposed to shot noise-limited) detection scheme, it is possible to anticipate less than 1 detected photon for each accumulation in the dot product computation, for sufficiently large vector size.

Through a combination of the optimization measures mentioned above, the energy consumption for optical fan-in can be reduced to the level of 100 aJ per addition, which is substantially more efficient than digital electronic implementation of accumulation operations (e.g., an 8-bit digital addition with two operands costs ~ 10 fJ [13], and reading/writing each intermediate partial sum costs additional 10s fJ, at least).

The energy analysis of the optical fan-in operation can be generalized beyond the accumulation of incoherent spatial modes. When multiple *frequency* or *temporal* modes are impinging onto the same detector, the total optical energy measured by the detector still equals the sum of the optical energy in each individual (frequency or temporal) mode [10, 12, 14–16], even if coherent light is used — this is due, of course, to energy conservation. More concretely, the energy of frequency and temporal modes can sum up in the same fashion as incoherent spatial modes, because light pulses of different wavelengths or pulses arriving at different times at the same detector do not interfere with each other. In principle, the energy cost associated with summing temporal or frequency modes should be no more than summing spatial modes, since the detector size (capacitance, and thermal noise) no longer needs to scale with the number of modes, as discussed before in this section. More detailed discussion on the energy scaling on these different ONN platforms can be found in, for example, Ref. [10–12].

Supplementary Note 10. RELATION TO STANFORD MATRIX-VECTOR MULTIPLIER

Our vector-vector dot product multiplier can be expanded into a full matrix-vector multiplier by the addition of optical fan-out operation. Such a matrix-vector multiplier can be viewed as a generalization of the classical Stanford matrix-vector multiplier, whose operation can be summarized in three steps: optical fan-out, element-wise multiplication, and optical fan-in (Supplementary Figure 12). The major difference between the proposed scheme and the Stanford matrix-vector multiplier is that the input vector and its corresponding weights (the weight vector) are geometrically arranged in 2D blocks instead of 1D arrays, which lead to different physical implementations. Compared to 1D arrays, we were motivated to employ the 2D-block arrangement for both input and weight vectors in this study, since it allows larger vector sizes to be used for vector-vector dot products.

Purely optical fan-out is necessary to achieve additional energy saving for optical matrix-vector multiplication, in comparison to performing vector-vector dot products sequentially one by one. This is because the energy for encoding input vectors can be amortized by making optical instead of digital copies of the same vector. The optical fan-out operation would allow a very low number of photons in each spatial mode: the optical fan-out splits the photons emitted from a light source and distribute each portion of the photons into many spatial modes, with each mode performs a scalar multiplication. In addition, since each spatial mode after optical fan-in is the result of many vacuum modes being coupled to the original spatial mode populated by a single light source, the noise of each spatial mode can be reduced to be quite close to shot noise, even if the light source intensity is not perfectly stable.

In the classical Stanford matrix-vector multiplier both optical fan-out and fan-in are implemented with cylindrical lenses as reverse processes of each other. In the 2D-block arrangement, this symmetry is broken because the optical fan-out process needs to create copies of the same block, while the optical fan-in focuses all elements in each block. For the 2D-block scheme, it is possible to implement optical fan-out with several techniques, including imaging with a microlens array [17, 18] or beam splitting using an array of beam splitters [19]. The concept of optical fan-out in

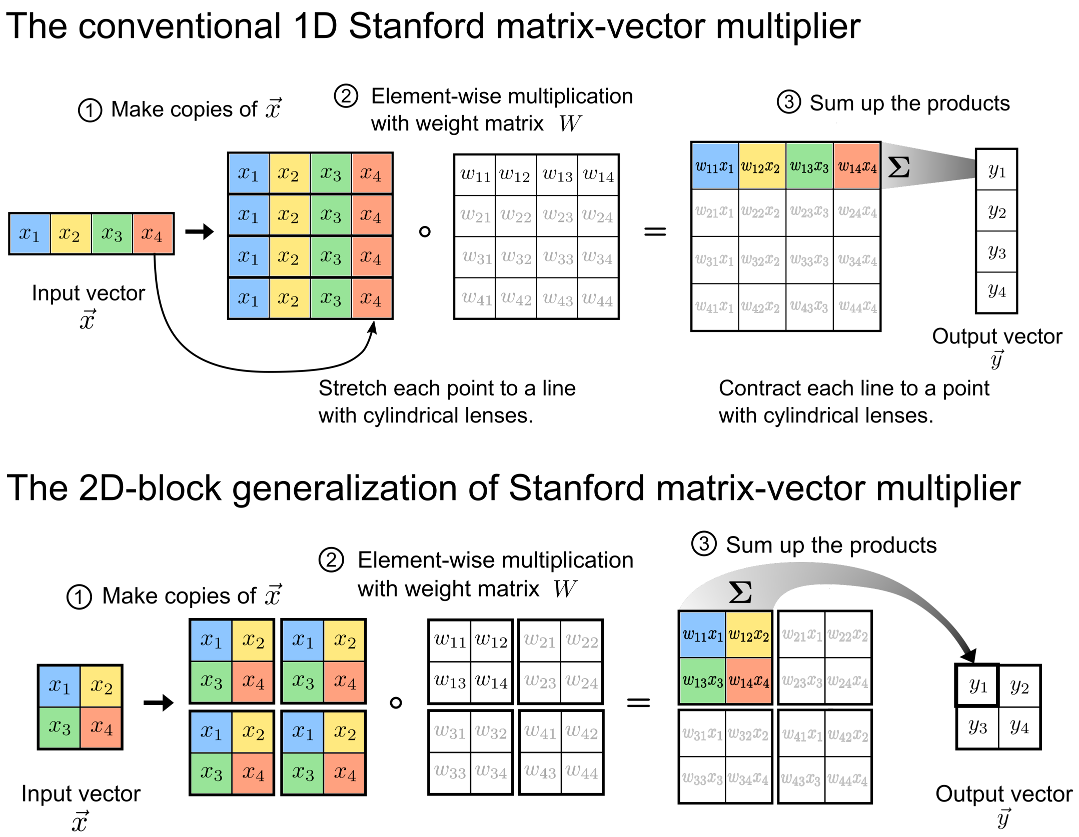

Supplementary Figure 12. The Comparison between the Classical Stanford Matrix-vector Multiplier and our 2D-block Scheme. The top row shows how the classical Stanford Matrix-vector Multiplier performs matrix-vector multiplication in a sequence of three steps of optical fan-out, element-wise multiplication, and optical fan-in. The bottom row shows how the 2D-block scheme performs the same corresponding operations.

spatial-domain ONNs is analogous to the concept of broadcasting on integrated-photonic platforms, which can be implemented straightforwardly through fiber [15] or waveguide [16, 20] couplers.

Part II

Vector-Vector Dot Product Accuracy

We characterized the precision of our optical matrix-vector multiplication by computing vector-vector dot products, which constitute general matrix-vector multiplication. The answer y (scalar) to the dot product between *input vector* $\vec{x}$ and *weight vector* $\vec{w}$ is defined as:

$$y = \vec{w} \cdot \vec{x} = \sum_{k=1}^{N} w_k x_k. \tag{1}$$

where x_k, w_k are in general real numbers.

Supplementary Note 11. COMPUTING DOT PRODUCTS WITH SIGNED ELEMENTS USING INCOHERENT LIGHT

Our setup can only perform dot products between vectors with non-negative elements, because x_k is encoded with the intensity of each spatial mode, and w_k is encoded with the transmission of each spatial mode. However, dot products between vectors of signed elements can always be reduced to those between non-negative-valued vectors with minimal digital processing overhead [2]. For a general vector with signed elements $\vec{x}^{\text{signed}}$, where each element $x_k^{\text{signed}} \in [x_{\text{min}}^{\text{signed}}, x_{\text{max}}^{\text{signed}}]$, $x_{\text{min}}^{\text{signed}}, x_{\text{max}}^{\text{signed}} \in \mathbb{R}$, a non-negative vector $\vec{x}'$ can be obtained by adding bias terms and rescaling:

$$\vec{x}' = \frac{x'_{\text{max}} - x'_{\text{min}}}{x_{\text{max}}^{\text{signed}} - x_{\text{min}}^{\text{signed}}}\, \vec{x}^{\text{signed}} + \frac{x_{\text{max}}^{\text{signed}} x'_{\text{min}} - x_{\text{min}}^{\text{signed}} x'_{\text{max}}}{x_{\text{max}}^{\text{signed}} - x_{\text{min}}^{\text{signed}}}\, \vec{1}, \tag{2}$$

where $\vec{1} = (1, 1, \cdots, 1)$ is a constant vector of size N. It can be verified that x'_k is tightly bounded between $[x'_{\text{min}}, x'_{\text{max}}]$ with $x'_{\text{max}} \geq x'_{\text{min}} \geq 0$. Therefore, two vectors with signed elements $\vec{w}^{\text{signed}}$ and $\vec{x}^{\text{signed}}$ can be converted to non-negative vectors $\vec{w}'$ and $\vec{x}'$, and the dot product $\vec{w}^{\text{signed}} \cdot \vec{x}^{\text{signed}}$ equals to the linear combination of $\vec{w}' \cdot \vec{x}'$, $\vec{w}' \cdot \vec{1}$, $\vec{1} \cdot \vec{x}'$ and a constant term. All three dot products are between vectors of non-negative elements. The dot product between $\vec{1}$ and any vector equals the summation of all of the vector elements, which were computed optically like any other dot product.

In machine learning applications, the input vector $\vec{x}'$ is either the input to the neural network or the neural activation of the previous layer, both of which are non-negative values after the ReLU nonlinear activation function. Therefore, since $\vec{x}^{\text{signed}}$ is already non-negative with $x_{\text{min}}^{\text{signed}} = 0$,

$$\vec{w}^{\,\text{signed}} \cdot \vec{x}^{\,\text{signed}} = c_1\, \vec{w}' \cdot \vec{x}' + c_2\, \vec{1} \cdot \vec{x}'. \tag{3}$$

For simplicity, both $\vec{x}'$ and $\vec{w}'$ can be normalized to the range $[0, 1]$, and the coefficients in Eq. 3 can be solved as: $c_1 = (w_{\text{max}}^{\text{signed}} - w_{\text{min}}^{\text{signed}}) x_{\text{max}}^{\text{signed}}$, $c_2 = w_{\text{min}}^{\text{signed}} x_{\text{max}}^{\text{signed}}$. The normalized vectors $\vec{x}'$ and $\vec{w}'$ were loaded onto the OLED display and the SLM, respectively, according to the hardware LUTs (e.g., Supplementary Figure 3c and Supplementary Figure 4b). In reality, the SLM could not achieve zero transmission which led to a minimum modulation $x_{\text{min}}^{\text{signed}} = \epsilon = 0.02$ (Supplementary Figure 4b). In other words, w'_k was normalized to the range $[\epsilon, 1]$ instead of $[0, 1]$. In this case, $c_1 = \frac{1}{1-\epsilon}(w_{\text{max}}^{\text{signed}} - w_{\text{min}}^{\text{signed}}) x_{\text{max}}^{\text{signed}}$, $c_2 = \frac{1}{1-\epsilon}(w_{\text{min}}^{\text{signed}} - \epsilon w_{\text{max}}^{\text{signed}}) x_{\text{max}}^{\text{signed}}$.

In summary, the dot product between two vectors with signed elements can be converted to two dot products between non-negative vectors, which can be first computed purely with optics, and then combined with only 2 digital multiplications and 2 digital additions. In other words, the price of conversion is a doubling of the amount of optical computation with a constant digital overhead, independent of vector size N.

For matrix-vector multiplication, the computational overhead can be further reduced, since $\vec{1} \cdot \vec{x}'$ remains the same for the dot product between $\vec{x}$ and any row vector of the matrix. As a result, $\vec{1} \cdot \vec{x}'$ only needs to be computed once optically and can be reused afterwards. In other words, to compute a matrix of size $N' \times N$ multiplied with a vector of size N, in addition to the N' optical dot products (which constitute NN' MACs), only one additional optical dot product ($\vec{1} \cdot \vec{x}'$) is required. Thus, the amount of digital overhead is on the order of $O(N')$.

Supplementary Note 12. CHARACTERIZATION OF DOT PRODUCT ACCURACY WITH VARYING PHOTON BUDGET

A. Generation of Test Datasets

To generate a test dataset representative of general dot products, we randomly generated vector pairs $\vec{x}$ and $\vec{w}$ based on natural scene images from the STL10 dataset. Each vector was generated from a single color channel of one or more images patched together, depending on the target vector size (each image of size $L \times L$ contributes $N = L^2$ elements to the vector). We chose natural images since they are more representative of the inputs in image classification with globally inhomogeneous and locally smooth features. To adjust the sparsity of the vectors, different thresholds were applied to the image pixel values such that the dot product results cover a wider range of possible values. This was achieved by shifting the original pixel values (float point numbers normalized to the range 0-1) in the entire image up or down by a certain amount, unless the value was already saturated at 1 (the maximum) or 0 (dark). For example, a shift of -1 would make the whole image dark. A shift of +0.2 would make all the pixel values that were originally larger than 0.8 saturated, and would increase all other pixel values by 0.2. This method allowed us to tune the overall intensity of the modulated images without losing the randomness of the distribution.

The computation of dot products on the setup followed the same steps of element-wise multiplication and optical fan-in, as described in the main text. Supplementary Figure 13 shows a few more examples of element-wise multiplication, similar to Figure 2a in the main text.

B. Data Collection Scheme and Photon Budget Control

In order to study how dot product accuracy changes with photon budget, we used a sensitive detector (MPPC) to measure the integrated optical energy. The optical energy consumed for each dot product computation was controlled by tuning the detector integration times (e.g., Supplementary Figure 10 had an integration time of 150 ns). To get enough statistics for noise distribution under low optical power, each detector readout measurement was repeated T times for each vector pair $\vec{w}$ and $\vec{x}$. To get error statistics representative of general vector pairs, we also repeated the measurement for S randomly generated vector pairs of different sparsity from randomly chosen images, as discussed in Supplementary Note 12 A and Supplementary Figure 13). We call this set of vector pairs the calibration dataset, and collected a total of $S \times T$ data points. Detector readout $v_{i,j}$ denotes the jth ($j = 1, 2, \ldots, T$) measurement made on the ith ($i = 1, 2, \ldots, S$) vector pair. For each vector pair, the mean value of the detector readouts $\bar{v}_i = 1/T \sum_{j=1}^{T} v_{i,j}$ was calculated for large enough T to eliminate the noise. The detector readouts were quantified either in optical energy, or in number of photons, which is optical energy divided by the photon energy (i.e., ∼0.4 aJ at 525 nm). To enable energy efficiency comparisons between different vector sizes, the total optical energy, or number of photons,

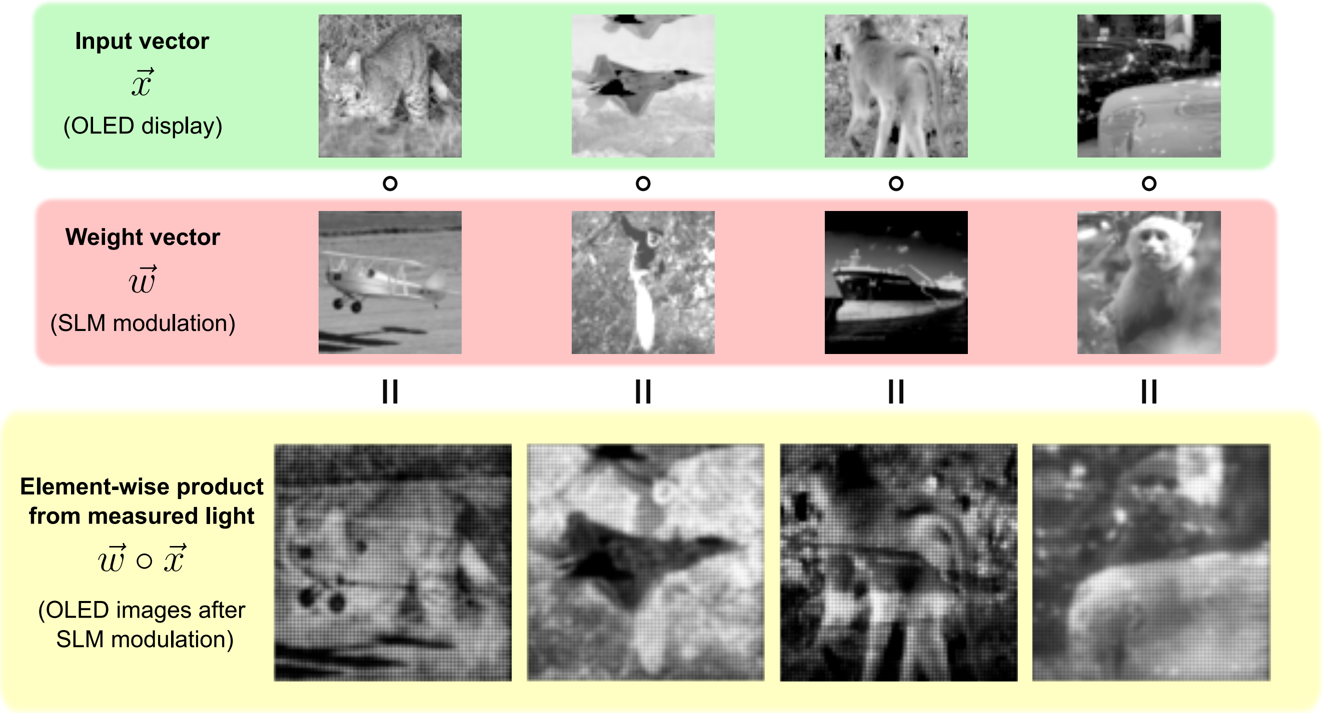

Supplementary Figure 13. Example Measurement of Element-wise Multiplication between Random Vectors. The top two rows show the corresponding input vectors on the OLED display and the weight vectors on the SLM. The bottom row displays modulated light captured by a camera. The input and weight vectors were generated from images in the STL10 dataset of size 64×64 (or $64 \times 64 = 4,096$ elements). Individual pixels are visible in the captured images.

detected for each dot product was further divided by the number of multiplications in the dot product.

C. Calibration of Detector Readouts

A calibration model f was made to convert the average detector readout $\bar{v}_i$ to the dot product result $y_{\text{meas},i}$ as $y_{\text{meas},i} = f(\bar{v}_i)$. The calibration involved plotting the ground truth of the dot product $y_{\text{truth},i} = \vec{x}_i \cdot \vec{w}_i$ versus $\bar{v}_i$, followed by fitting the data points to a linear curve using a least-squares criterion. Supplementary Figure 14 shows an example of data points measured on vector pairs of length $N = 505521$. The calibration curve f is plotted in the dashed red line. The range of y_{truth} was normalized to $[0, 1]$ by rescaling $\vec{x}'$ and $\vec{w}'$, based on their definitions in Eq. 2, with a multiplicative factor $1/\sqrt{N}$. With the calibration model, we could read out the dot product result based on the detector readout value. In principle, the calibration only needed to be performed once with the calibration dataset, unless the setup changed (e.g., adding extra attenuation) or has drifted over time.

D. Quantification of Single-Shot Dot Product Computation Error

After obtaining the calibration curve, we generated another random vector pair test dataset in order to quantify the error statistics of dot product computation performed by our setup. Error was defined as the difference between the measured result and ground truth $y_{\text{truth}} - y_{\text{meas}}$. Suppose we have S vector pairs in the data set and each is

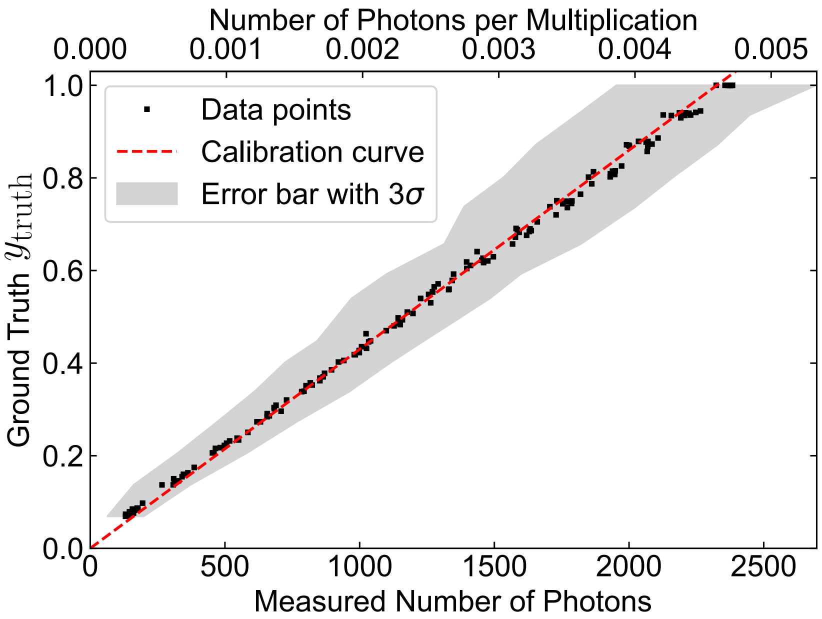

Supplementary Figure 14. A Calibration Curve Converting Detector Readouts to Dot Product Results. The mean detector readout ($\bar{v}_i$, x axis) is plotted against the corresponding ground truth ($y_{\mathrm{truth},i}$, y axis) for every vector pair in the calibration dataset. The unit of detector readout is either the number of photons (bottom axis) or the number of photons per multiplication (top axis). The vector length was 711×711 ($N = 505{,}521$). The calibration curve (red dashed line) was obtained using a least-squares fit to the data points. The shaded area indicates 3 standard deviations of the repeated measurements. The average number of photons per multiplication of the data points in the plot is 0.0025.

repeated T times. For each detector readout $v_{i,j}$ $(i = 1, 2, \ldots, S,\ j = 1, 2, \ldots, T)$, $\mathrm{Error}_{i,j} = y_{\mathrm{truth},i} - y_{\mathrm{meas},i,j}$, where $y_{\mathrm{meas},i,j} = f(v_{i,j})$. Unlike calibration, here we used single-shot readouts $v_{i,j}$ rather than the mean value $\bar{v}_i$.

For each vector pair $\vec{x}_i$ and $\vec{w}_i$, the root-mean-square (RMS) error for different measurement trials was calculated as $\mathrm{RMSE}_i = \sqrt{\frac{1}{T}\sum_{j=1}^{T} \mathrm{Error}_{i,j}^2}$, which can be interpreted roughly as the most likely error one would get from a single-shot computation for vector pair index i. The total RMS error across different vectors in the dataset was calculated as $\mathrm{RMSE} = \sqrt{\frac{1}{S}\sum_{i=1}^{S} \mathrm{RMSE}_i^2} = \sqrt{\frac{1}{ST}\sum_{i,j} \mathrm{Error}_{i,j}^2}$, which could be interpreted as the most likely error one would get from a single-shot computation by randomly selecting a vector pair from the entire test dataset. Histograms of the errors of the test dataset are shown in Supplementary Figure 15 insets.

The scatter plots in Supplementary Figure 15 show how well the computed dot product results matched the ground truth, under different photon budgets. The average number of photons detected during these experiments were different, since they were determined by detector integration time. With higher photon budgets, the error decreases as the noise contribution to the error decreases. For a higher photon budget (>1 photon per multiplication), the RMS error stops decreasing and is instead limited by systematic error due to imperfections in the setup. The four scatter plots in Supplementary Figure 15) correspond to the total RMSE data point of the same color in Figure 2b in the main text.

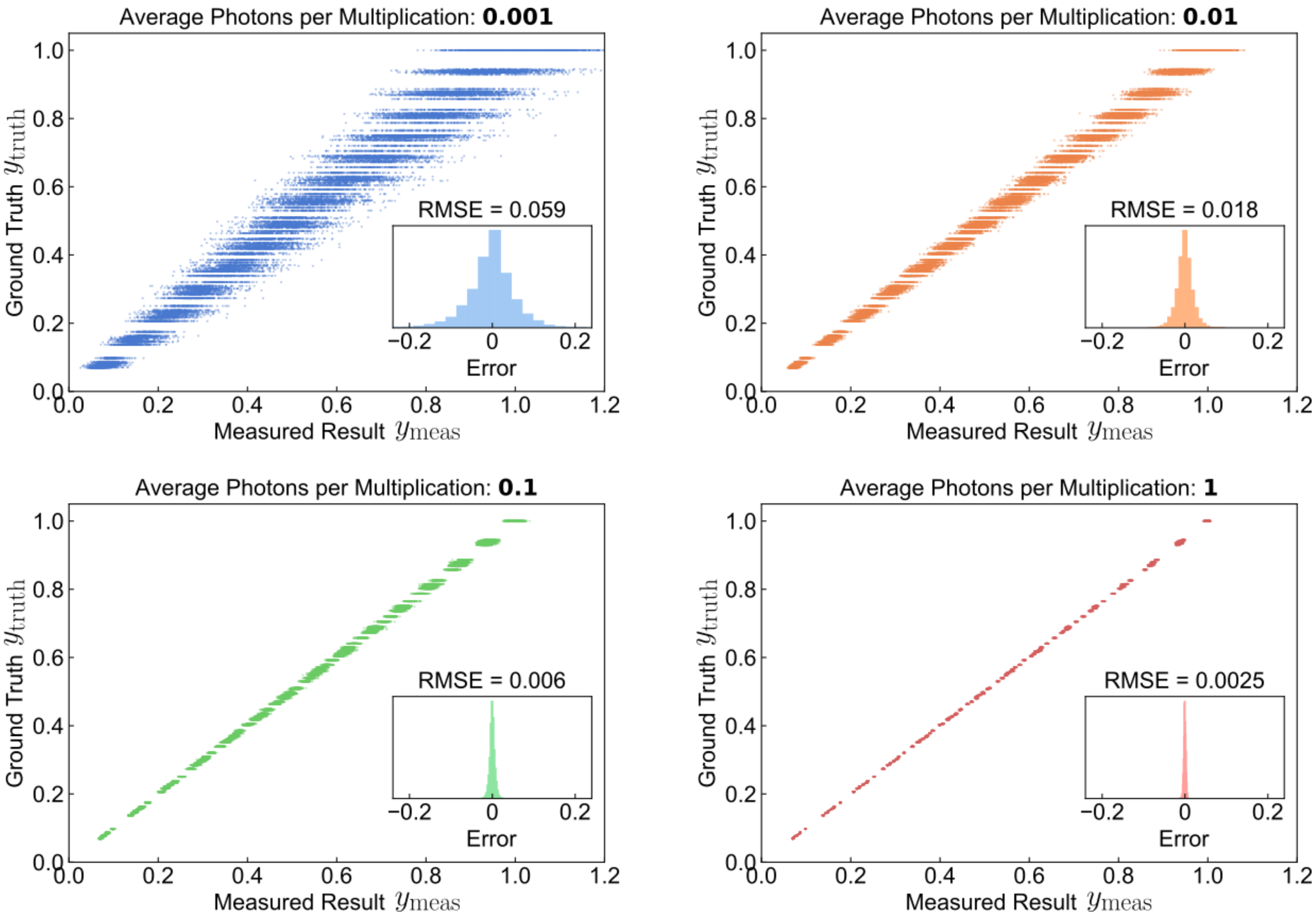

Supplementary Figure 15. Dot Product Error Analysis at 4 Typical Photon Budgets. The dot products were computed with vector size $N = 711 \times 711 = 505,521$). The average number of photons per multiplication (indicated at the top of each plot) was controlled by the integration time of the MPPC detector, and averaged over the entire test dataset. For each vector pair, the measurement $y_{\text{meas},i,j}$ was repeated multiple times, and all the data points were plotted. The ground truth $y_{\text{truth},i}$ is plotted against the corresponding measurements $y_{\text{meas},i,j}$. The histogram of errors $y_{\text{truth},i} - y_{\text{meas},i,j}$ is shown in each inset. The overall accuracy representative of the dataset is characterized by the root-mean-square error (RMSE), which is also similar to the value of the standard deviation of the error distribution. The color code is the same as that in Figure 2 in the main text.

Part III

Optical Neural Network for Image Classification

Supplementary Note 13. TRAINING PROTOCOL OF NOISE RESILIENT OPTICAL NEURAL NETWORKS

For handwritten digit classification (MNIST database), we trained a 4-layer neural network with full connections (i.e., a multilayer perceptron, MLP). The inputs are 8-bit grayscale images of size $28 \times 28 = 784$ total pixels, followed by two fully-connected hidden layers, each comprising 100 neurons with ReLU as the nonlinear activation function. The output layer has 10 neurons, with each neuron corresponding to a digit from 0 to 9. The neural network was implemented and trained in PyTorch (1.7.0). To improve the robustness of the model to numerical error, we employed several techniques during training:

1. Quantization-aware training (QAT): The activation of neurons were quantized to 4 bits and weights to 5 bits to adapt to the numerical precision of the setup. For example, for vector size of 784, we found the SNR of dot products is equivalent to ∼4-bit numerical precision. Even though the SLM could be controlled with 8-bit numbers, we decided to quantize its weight to 5 bits, which matched better with its extinction ratio of 50 and did not seem to have any negative impact on MLP accuracy. To reduce the numerical sensitivity caused by quantization (e.g., in the regular nearest neighbor scheme, 0.49 is rounded down to 0, while 0.51 is rounded up to 1), we used random digitization between the adjacent levels, which was observed to improve the model robustness against random noise. We found that a few (6-12) warm-up training epochs with full 32-bit float precision helped to protect the model from aggressive quantization in the initial stage, after which the application of quantization noise was less likely to derail the training process, but still helped to fine-tune the parameters.

2. Data augmentation with random image transform and convolution: To improve model tolerance to potential hardware imperfections, we imitated similar kinds of errors on input images. For example, the misalignment was modeled as random rotation (within $\pm 5°$) and translation ($\pm 4\%$ of image size in any direction), mismatched zoom factor as random zooming ($\pm 4\%$ of image size), and intra-pixel crosstalk as a mild convolution with a 3×3 blurring kernel. We observed that these measures not only helped to improve model immunity to imaging error, but also improved overall model accuracy and robustness to photon noise by reducing overfitting with regularization. The data augmentation was only performed on the input layer rather than all hidden layers, due to computational complexity and the observation that hidden layers were usually more sparse, making crosstalk between neighboring pixels was less of an issue.

3. Optimizing training parameters: We used a stochastic gradient optimizer for training with a learning rate typically between 0.03 and 0.05, and a momentum between 0.7 and 1. Learning rate decay was applied every 20 epochs with a decaying rate between 0.3 and 0.5. The training parameters were randomly generated within the range for different trials of training, and fine-tuned by using the package Optuna [21].

The training of each model took 100 epochs. Several hundred models were trained, each with slightly different randomly generated training parameters. Afterwards, each model was executed with simulated shot noise at different photon budgets (code available at: `https://github.com/mcmahon-lab/ONN-QAT-SQL`), and then the model yielding the best accuracy at low photon budgets was chosen to be run on the ONN setup.

It is important to note that the quantization of neuron activations was only performed during training on a digital computer, but not during the inference stage on the ONN. We observed that even though the models were trained with noise, they still performed better when run with full precision. Therefore, the trained weights and neural activations

were loaded with the maximum allowable precision for the hardware (i.e., 7 bits for the OLED display, and 8 bits for the SLM). The training code can be found in this GitHub repository: `https://github.com/mcmahon-lab/ONN-QAT-SQL`.

The neural network model was completely trained on a digital computer, without any customization to the optical setup. There was no *in situ* re-training of any model parameter, nor any addition of extra digital layers to assist with the optical setup. To be clear, such digital assistance is extremely useful for bridging the gap between hardware and software in real-world applications. However, they were not used for this study because they would have affected the one-to-one correspondence between optical and digital operations, and therefore our ability to unambiguously quantify the number of optical operations — and the amount of optical energy used — for each optical operation.

Supplementary Note 14. WORKFLOW FOR RUNNING OPTICAL NEURAL NETWORKS FOR INFERENCE

The trained neural network (MLP 784-100-100-10 as described in Supplementary Note 13) was executed for inference with our optical vector-vector dot product multiplier in the following steps:

1. Starting from the input layer, the matrix-vector multiplication involved in the forward propagation from the current layer to the next layer was computed optically by each dot product, according to the procedure described in Supplementary Note 11. The matrix weights loaded onto the SLM were exactly the same as those in the neural network trained on the digital computer. The number of photons per multiplication in matrix-vector multiplication was controlled by adjusting the number of detector samples to sum.

2. The bias terms and the nonlinear activation function were applied digitally to the matrix-vector multiplication result, and these parameters followed exactly as those in the trained neural network, without any modification or retraining. The resulting neuron activations were used as the input vector to the matrix-vector multiplication that leads to the next layer (go back to step 1) unless there is none (go to step 3).

3. At the output layer, the prediction was made based on the highest score.

For step 1, since the inputs and neural activations were both non-negative due to our choice of ReLU nonlinearity, only the weight matrices needed to be shifted and normalized. The element-wise multiplication of the first layer is visualized in Supplementary Figure 16b, with the weight matrix displayed on the SLM visualized in Supplementary Figure 16a for comparison. Each matrix-vector multiplication $y_i = \sum_j W_{ij} x_j$ was decomposed into vector-vector dot products $\vec{w_i} \cdot \vec{x}$, where $\vec{w_i} = W_{ij}$ $(j = 1, 2, ...)$ encodes the (synaptic) weights from neuron i in the previous layer to neuron j in the next layer, and $\vec{x} = x_j$ $(j = 1, 2, ...)$ encodes the neural activations of the previous layer. Each modulated handwritten-digit image shown in Supplementary Figure 16b illustrates the element-wise product $\vec{w_i} \circ \vec{x}$. To obtain the answer to the matrix-vector multiplication, element-wise modulated spatial modes in each block (i.e., $\vec{w_i} \circ \vec{x}$) were summed up by optical fan-in as described in Supplementary Note 9, which is equivalent to summing all the pixels in each block shown in the image taken by the camera in Supplementary Figure 16. The degree of optical fan-in equals the vector size or the number of neurons in the previous layer (i.e., 784 for the first layer, 100 for the second layer etc.). The $\vec{1} \cdot \vec{x} = \sum_j x_j$ term in Eq. 3 was computed by adding an additional input vector block and setting the corresponding SLM pixels' transmissivity to the maximum in order to encode $\vec{1}$ (e.g., Supplementary Figure 16b, last row in the image. An entire row was used for illustration purposes and redundancy, while in fact only one additional block was needed for the entire layer.). The integrated optical energy was translated to the answer of the dot product based on a calibration curve, which was made by fitting the measured optical energy to the ground truth of the dot products using the first 10 samples of the MNIST test dataset in a fashion similar to that described in Supplementary Note 12 C.

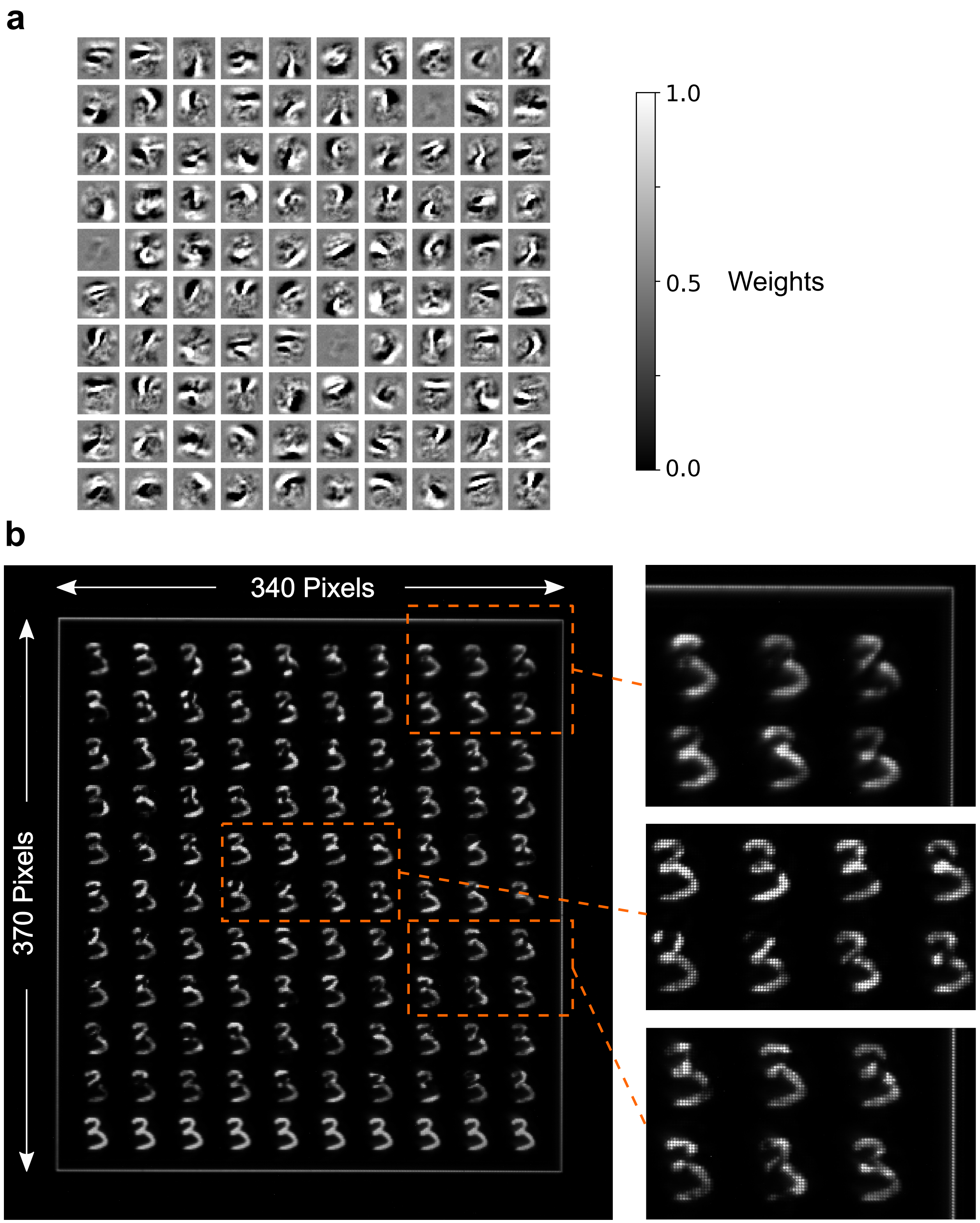

Supplementary Figure 16. An Example of the Matrix-vector Multiplication Results of the First Fully-connected Layer of the ONN. a, Visualization of the weights of the connection between the first and the second layer of neurons; the pixel values in each square array indicate the weights from all the neurons in the first layer to one of the neurons in the second layer of neurons. The weights were implemented as light transmission on the SLM (1 for full light transmission and 0 for no light transmission.). **b,** The images show the results of element-wise multiplication between an input vector ($28 \times 28 = 784$ elements) and each row of the matrix of the first fully-connected layer (100×784) of our ONN model, as captured by a camera. The last row computed $\sum_j x_j$, which was used to offset the output vector for negative elements. Only the output of the first layer is shown.

Supplementary Note 15. ENERGY EFFICIENCY OF THE OPTICAL NEURAL NETWORK

The total number of optical operations is calculated as the number of operations involved in each matrix-vector multiplication from one neural network layer to the next. For an input layer of width N (also referred to as N neurons in this layer) and an output layer width N', the total number of scalar multiplication equals NN' and the total number of scalar addition is $(N-1)N'$. According to Supplementary Note 14, the four layers of the ONN have width 784, 100, 100, and 10, respectively. For each forward propagation through the neural network, the total number of optical multiplications — defined as scalar multiplication between a pair of non-negative real numbers — is $784 \times (100+1) + 100 \times (100+1) + 100 \times (10+1) =$ **90,384** (the additional +1 term in each output layer width is due to the calculation of one additional dot product $\vec{1} \cdot \vec{x}$ as an offset term, see Supplementary Note 14). The total number of optical additions, defined as the addition of a pair of non-negative numbers, is $(784-1) \times (100+1) + (100-1) \times (100+1) + (100-1) \times (10+1) =$ **90,171**. The total digital electronic operations for each forward propagation includes $100 + 100 + 10 = 210$ 16-bit additions for applying the $\vec{1} \cdot \vec{x}$ term (to shift the dot product between vectors of non-negative elements in order to obtain the dot product between vectors of signed elements), $100+100+10 = 210$ 16-bit additions for applying digital biases, and $100+100 = 200$ applications of ReLU nonlinear activation functions (involving, essentially, a comparison between each element and 0, and then setting the element to 0 if it is less than 0). The total count of all operations, including optical and electronic, for a single forward pass of the neural network, is summarized in Supplementary Table 1.

Optical operations account for $100\% - 621/180,555 = 99.66\%$ of the total amount of operations involved in a single forward pass of the neural network to classify a full-resolution 28×28 handwritten-digit image (also referred to as per inference). More importantly, we did not use any additional trainable digital layer to read out results from or adapt to the physical setup. This practice is necessary for the accurate quantification of optical-operation energy efficiency. It is a well-known fact in machine learning that a single linear layer can already achieve 91.6% classification accuracy on the benchmark dataset of MNIST (`http://yann.lecun.com/exdb/mnist/`), and a straightforward implementation of reservoir computer or extreme learning machine with a *random* neural network and a single digital trainable output layer can achieve even 98% accuracy [22]! In the case of optical reservoir computing, the optical layer performs a huge amount of computation with high efficiency, but the computation is almost random and has limited programmability. As a result, a digital output layer is required to extract useful results from optical computation that are relevant to the task at hand. In order to isolate the contributions of digital electronic operations from those of optical operations, we restrained ourselves from using any trainable digital operations to adapt to the experimental setup in this work. The neural network model run on the ONN setup was isomorphic [23] to the one run on a digital computer: each optical operation replaced a corresponding digital operation in the trained neural network model that could otherwise be executed on a digital computer, without changing any parameter (e.g., matrix weights). The absence of any extra trainable digital parameter forced the ONN to solely rely on its optical operations: each optical operation needed to be engineered to be highly effective and faithful to the neural network model in order to contribute to the correct classification result.

The optical energy per operation was quantified according to the following scheme: after optical fan-in, all the spatial modes involved in a vector-vector dot product were focused on a bucket detector (MPPC). The detected optical energy per dot product was measured by integrating optical power over a period of time. The number of photons detected per multiplication was then calculated as:

$$\#\text{ of photons detected per multiplication} = \frac{\text{Detected optical energy per dot product}}{\#\text{ of multiplications per dot product} \times \text{Single photon energy at } 525\,\text{nm}}$$

The total detected optical energy for each layer during the execution of the ONN for one inference was calculated with the following equation:

$$\text{Total detected optical energy per layer} = \text{Detected optical energy per dot product} \times \#\text{ of dot products per layer}$$

The integration time of the detector was typically chosen in the range of 1 µs to 60 µs, in order to keep the number of photons per multiplication approximately constant across different layers. A few different values of the number of photons per multiplication were chosen in order to observe how the classification accuracy of the ONN changes with photon budget. The top section of Supplementary Table 2 shows the average detected optical energy per dot product for each layer of the ONN. The middle section of Supplementary Table 2 shows the detected photons per multiplication by each layer of the ONN, including a weighted average across the entire ONN, which is also plotted in Figure 3b in the main text. The weighted averages were derived by weighting with the total number of multiplications in each layer (listed in Supplementary Table 1). The bottom section of Supplementary Table 2 shows the total detected optical energy for each layer and the entire ONN during one inference, which is also plotted against the top horizontal axis in Figure 3b in the main text.

When studying the energy efficiency of ONNs, attention should also be paid to the total number of optical operations required to achieve certain level of performance on the benchmark task, in addition to the energy efficiency of each operation [24]. This is because the number of operations of different neural network models to solve the same benchmark problem is generally different, but ultimately the energy efficiency should be determined by the *total* amount of energy to solve the problem instead of the energy consumption of each operation. Take MNIST as an example again, $\sim 10^6$ digital operations are regularly needed to reach $98 \sim 99\%$ classification accuracy with multi-layer perceptrons (`http://yann.lecun.com/exdb/mnist/`). The total number of optical plus electronic operations shown in this work (Supplementary Table 2) falls well within this range. This indicates that the high optical energy efficiency per operation shown in this work readily translates to a high optical energy efficiency per inference (see Supplementary Table 2 bottom section), after multiplying with the total number of operations in the neural network model. The 100-fJ level detected optical energy per inference is several orders of magnitude smaller than the total energy consumption per inference of digital electronic processors that are highly optimized for energy efficiency on the same benchmark tasks (10^3 times smaller than Ref. [25] with binary weights and activations, $\sim 10^5$ smaller than Ref. [26, 27]). Admittedly, once the optical loss and non-optical energy is included in the total energy consumption of ONNs, the gap between the energy consumption of optical and digital electronic processing would decrease. Nevertheless, the low detected optical energy per inference shown in this work indicates optical processors have the potentials to achieve quite large advantage over electronic processors in terms of the whole-system energy efficiency provided that the entire system including optical and non-optical parts are sufficiently optimized.

The power consumption of each experimental instrument is listed in Supplementary Table 3 for the completeness of experimental details. Note that the *whole-system* energy efficiency derived from these power consumption does *not* carry any significant implication on the overall energy efficiency of the ONN, since none of the devices was customized for optimized power consumption or refresh rate. For one example, based on the amount of digital operation needed, the laptop can be probably replaced by a digital processor that consumes orders of magnitude lower power. For another example, the maximum data throughput rate in this experiment was limited by the refresh rate of OLED at 60 Hz, which was made to adapt to human eye, most likely the speed can be increased several orders of magnitude as well if the device were optimized for optical computing purposes. Due to the large non-optical energy consumption overhead in the hardware that is irrelevant to the ONN computation, the total power consumption of the setup was constant at ~65 W (Supplementary Table 3), regardless of the speed of computation. The maximum achievable computational speed is (711^2 multiplications/frame + ($711^2 - 1$) additions/frame) × (60 frames/second) $\sim 6.07 \times 10^7$ operations/s. At this computing rate, the energy efficiency is (6.07×10^7 operations/s)/65 W $= 0.93 \times 10^6$ operations/J. Since the experimental setup was built for the purpose of studying the optical energy efficiency of ONNs with large-scale optical dot products, rather than as a full engineering solution optimized for competitive performances, the computational speed and energy efficiency reported above should be interpreted as experimental conditions instead of as the specification of a final engineering product. For interested readers, a comparison of the computational speed and energy efficiency of several cutting-edge ONNs can be found in Ref. [6].

Supplementary Table 1. The number of all operations during the execution of the ONN for one inference

	Layer1 → 2 (784-100)	Layer2 → 3 (100-100)	Layer3 → 4 (100-10)	Whole network
Number of input neurons (dot-product vector size)	784	100	100	
Number of output neurons (total number of dot products)	100+1	100+1	10+1	
Total optical multiplications	79,184	10,100	1,100	90,384
Total optical additions	79,083	9,999	1,089	90,171
Digital addition of the offset term $\vec{1} \cdot \vec{x}$ (FP16)	100	100	10	210
Digital addition of model biases (FP16)	100	100	10	210
Digital ReLU nonlinear activation functions	100	100	0	200
Digital max/softmax for a 10-D vector	0	0	1	1
**Total number of optical operations**	158,267	20,099	2,189	180,555
**Total number of digital operations**	300	300	21	621

Supplementary Table 2. Breakdown of the average detected optical energy consumption, by layer, during the execution of the ONN for one inference

**Photons (optical energy) per dot product**				
Layer 1 → 2 (784-100)	Layer 2 → 3 (100-100)	Layer 3 → 4 (100-10)		Classification accuracy (%)
26.7 (10.1 aJ)	3.2 (1.2 aJ)	3.4 (1.3 aJ)		11.5
126.3 (47.8 aJ)	15.9 (6.0 aJ)	16.1 (6.1 aJ)		53.8
253.5 (95.9 aJ)	32.0 (12.1 aJ)	31.5 (11.9 aJ)		70.0
520.7 (197 aJ)	62.6 (23.7 aJ)	64.0 (24.2 aJ)		90.0
2439 (923 aJ)	314.5 (119 aJ)	306.6 (116 aJ)		99.2

**Photons (optical energy) per scalar multiplication**				
Layer 1 → 2 (784-100)	Layer 2 → 3 (100-100)	Layer 3 → 4 (100-10)	Weighted average	Classification accuracy (%)
0.034 (0.013 aJ)	0.032 (0.012 aJ)	0.035 (0.013 aJ)	0.034 (0.013 aJ)	11.5
0.161 (0.061 aJ)	0.159 (0.060 aJ)	0.161 (0.061 aJ)	0.16 (0.06 aJ)	53.8
0.323 (0.122 aJ)	0.319 (0.121 aJ)	0.316 (0.120 aJ)	0.32 (0.12 aJ)	70.0
0.665 (0.252 aJ)	0.627 (0.237 aJ)	0.638 (0.241 aJ)	0.66 (0.25 aJ)	90.0
3.109 (1.176 aJ)	3.144 (1.190 aJ)	3.070 (1.162 aJ)	3.11 (1.19 aJ)	99.2

**Optical energy per inference**				
Layer 1 → 2 (784-100)	Layer 2 → 3 (100-100)	Layer 3 → 4 (100-10)	Whole network	Classification accuracy (%)
1.00 fJ	0.12 fJ	0.013 fJ	1.14 fJ	11.5
4.83 fJ	0.61 fJ	0.067 fJ	5.50 fJ	53.8
9.68 fJ	1.22 fJ	0.13 fJ	11.0 fJ	70.0
19.9 fJ	2.40 fJ	0.27 fJ	22.6 fJ	90.0
93.2 fJ	12.0 fJ	1.28 fJ	107 fJ	99.2

Supplementary Table 3. Power consumption of each experimental device

Device	Power (W)
OLED display	~ 0.5
SLM + HDMI driver	18.5
MPPC detector	1.1
Digital laptop computer	~ 45
**Total**	~ 65

Supplementary References

[1] I. R. Berchera and I. P. Degiovanni, Quantum imaging with sub-Poissonian light: challenges and perspectives in optical metrology. *Metrologia* **56**, 024001 (2019).

[2] J. W. Goodman, A. Dias, and L. Woody, Fully parallel, high-speed incoherent optical method for performing discrete Fourier transforms. *Optics Letters* **2**, 1–3 (1978).

[3] D. Psaltis, D. Brady, and K. Wagner, Adaptive optical networks using photorefractive crystals. *Applied Optics* **27**, 1752–1759 (1988).

[4] M. Miscuglio, Z. Hu, S. Li, J. K. George, R. Capanna, H. Dalir, P. M. Bardet, P. Gupta, and V. J. Sorger, Massively parallel amplitude-only Fourier neural network. *Optica* **7**, 1812–1819 (2020).

[5] J. Chang, V. Sitzmann, X. Dun, W. Heidrich, and G. Wetzstein, Hybrid optical-electronic convolutional neural networks with optimized diffractive optics for image classification. *Scientific Reports* **8**, 1–10 (2018).

[6] T. Zhou, X. Lin, J. Wu, Y. Chen, H. Xie, Y. Li, J. Fan, H. Wu, L. Fang, and Q. Dai, Large-scale neuromorphic optoelectronic computing with a reconfigurable diffractive processing unit. *Nature Photonics* **15**, 367–373 (2021).

[7] X. Lin, Y. Rivenson, N. T. Yardimci, M. Veli, Y. Luo, M. Jarrahi, and A. Ozcan, All-optical machine learning using diffractive deep neural networks. *Science* **361**, 1004–1008 (2018).

[8] V. Sze, Y.-H. Chen, T.-J. Yang, and J. S. Emer, Efficient processing of deep neural networks: A tutorial and survey. *Proceedings of the IEEE* **105**, 2295–2329 (2017).

[9] N. P. Jouppi, C. Young, N. Patil, D. Patterson, G. Agrawal, R. Bajwa, S. Bates, S. Bhatia, N. Boden, A. Borchers, R. Boyle, P.-l. Cantin, C. Chao, C. Clark, C. Coriell, M. Daley, M. Dau, J. Dean, B. Gelb, T. V. Ghaemmagham et al. In-datacenter performance analysis of a tensor processing unit. In *Proceedings of the 44th Annual International Symposium on Computer Architecture (ISCA)*, 1–12 (2017).

[10] R. Hamerly, L. Bernstein, A. Sludds, M. Soljačić, and D. Englund, Large-scale optical neural networks based on photoelectric multiplication. *Physical Review X* **9**, 021032 (2019).

[11] D. A. B. Miller, Attojoule optoelectronics for low-energy information processing and communications. *Journal of Lightwave Technology* **35**, 346–396 (2017).

[12] A. N. Tait, Quantifying power use in silicon photonic neural networks. *arXiv:2108.04819* (2021).

[13] M. Horowitz, Computing's energy problem (and what we can do about it). In *2014 IEEE International Solid-State Circuits Conference Digest of Technical Papers (ISSCC)*, 10–14 (2014).

[14] M. A. Nahmias, T. F. De Lima, A. N. Tait, H.-T. Peng, B. J. Shastri, and P. R. Prucnal, Photonic multiply-accumulate operations for neural networks. *IEEE Journal of Selected Topics in Quantum Electronics* **26**, 1–18 (2020).

[15] X. Xu, M. Tan, B. Corcoran, J. Wu, A. Boes, T. G. Nguyen, S. T. Chu, B. E. Little, D. G. Hicks, R. Morandotti, A. Mitchell, and D. J. Moss, 11 TOPS photonic convolutional accelerator for optical neural networks. *Nature* **589**, 44–51 (2021).

[16] J. Feldmann, N. Youngblood, M. Karpov, H. Gehring, X. Li, M. Stappers, M. Le Gallo, X. Fu, A. Lukashchuk, A. S. Raja, C. D. Wright, A. Sebastian, T. J. Kippenberg, W. H. P. Pernice, and H. Bhaskaran, Parallel convolutional processing using an integrated photonic tensor core. *Nature* **589**, 52–58 (2021).

[17] W. Andregg, M. Andregg, R. T. Weverka, and L. Clermont, Wavelength multiplexed matrix-matrix multiplier. (U.S. Patent No. 10,274,989). U.S. Patent and Trademark Office (2019).

[18] Y. Hayasaki, I. Tohyama, T. Yatagai, M. Mori, and S. Ishihara, Optical learning neural network using Selfoc microlens array. *Japanese Journal of Applied Physics* **31**, 1689 (1992).

[19] A. Hemmi, R. Mizumura, R. Kawanishi, H. Nakajima, H. Zeng, K. Uchiyama, N. Kaneki, and T. Imato, Development of a novel two dimensional surface plasmon resonance sensor using multiplied beam splitting optics. *Sensors* **13**, 801–812 (2013).

[20] A. N. Tait, T. F. De Lima, M. A. Nahmias, H. B. Miller, H.-T. Peng, B. J. Shastri, and P. R. Prucnal, Silicon photonic modulator neuron. *Physical Review Applied* **11**, 064043 (2019).

[21] T. Akiba, S. Sano, T. Yanase, T. Ohta, and M. Koyama, Optuna: A next-generation hyperparameter optimization

framework. In *Proceedings of the 25th ACM SIGKDD International Conference on Knowledge Discovery and Data Mining* (2019).

[22] P. De Chazal, J. Tapson, and A. Van Schaik, A comparison of extreme learning machines and back-propagation trained feed-forward networks processing the mnist database. In *2015 IEEE International Conference on Acoustics, Speech and Signal Processing (ICASSP)*, 2165–2168 (2015).

[23] B. J. Shastri, A. N. Tait, T. F. de Lima, W. H. Pernice, H. Bhaskaran, C. D. Wright, and P. R. Prucnal, Photonics for artificial intelligence and neuromorphic computing. *Nature Photonics* **15**, 102–114 (2021).

[24] V. Sze, Y.-H. Chen, T.-J. Yang, and J. S. Emer, How to evaluate deep neural network processors: TOPS/W (alone) considered harmful. *IEEE Solid-State Circuits Magazine* **12**, 28–41 (2020).

[25] J. Zhang, Z. Wang, and N. Verma, A machine-learning classifier implemented in a standard 6T SRAM array. In *2016 IEEE Symposium on VLSI Circuits (VLSI-Circuits)*, 1–2 (2016).

[26] B. Moons, D. Bankman, L. Yang, B. Murmann, and M. Verhelst, BinarEye: An always-on energy-accuracy-scalable binary CNN processor with all memory on chip in 28nm CMOS. In *2018 IEEE Custom Integrated Circuits Conference (CICC)*, 1–4 (2018).

[27] J. Park, J. Lee, and D. Jeon, A 65-nm neuromorphic image classification processor with energy-efficient training through direct spike-only feedback. *IEEE Journal of Solid-State Circuits* **55**, 108–119 (2019).
